# Chemical Graph-Based Transformer Models for Yield Prediction of High-Throughput Cross-Coupling Reaction Datasets

**DOI:** 10.1021/acsomega.4c06113

**Published:** 2024-09-17

**Authors:** Akinori Sato, Ryosuke Asahara, Tomoyuki Miyao

**Affiliations:** † Data Science Center, Nara Institute of Science and Technology, 8916-5 Takayama-cho, Ikoma, Nara 630-0192, Japan; ‡ Graduate School of Science and Technology, Nara Institute of Science and Technology, 8916-5 Takayama-cho, Ikoma, Nara 630-0192, Japan

## Abstract

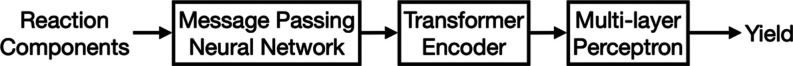

The chemical reaction yield is an important factor to determine the reaction conditions. Recently, many data-driven models for yield prediction using high-throughput experimentation datasets have been reported. In this study, we propose a neural network architecture based on the chemical graphs of the reaction components to predict the reaction yield. The proposed model is the sequential combination of a message-passing neural network and a transformer encoder (*MPNN-Transformer*). The reaction components are converted to molecular matrices by the first network, followed by the interplay of the reaction components in the second network after adding the embeddings of the compound roles in the chemical reaction. The predictive ability of the proposed models was compared with state-of-the-art yield prediction models using two high-throughput experimental datasets: the Buchwald–Hartwig cross-coupling (BHC) and Suzuki–Miyaura cross-coupling (SMC) reaction datasets. Overall, the *MPNN-Transformer* models showed high prediction accuracy for the BHC reaction datasets and some of the extrapolation-oriented SMC reaction datasets. These models also performed well when the training dataset size was relatively large. Furthermore, analyzing the poorly predicted reactions for the BHC reaction dataset revealed a limitation of the data-driven yield prediction approach based on the chemical structural similarity.

## 1 Introduction

The chemical reaction yield is defined as the number of moles of the product obtained divided by the theoretical number of moles of the product based on the stoichiometry, which is one of the most important factors to determine the reaction conditions. In other words, the reaction yield depends on the reaction conditions, such as temperature, concentrations, and reaction time. Thus, data-driven yield prediction using a dataset obtained from a homogeneous reaction condition is a reasonable approach,^1^ and several studies have been reported accordingly. Among the yield prediction models, machine learning (ML) including deep neural network models can predict the reaction yield with high accuracy when it is trained on a few thousand chemical reactions under controlled reaction conditions, which is called high-throughput experimentation (HTE).^2,3^ For example, highly predictive random forest (RF) models for the yield prediction of the Buchwald–Hartwig cross-coupling (BHC) reaction have been reported using a HTE dataset with the descriptors calculated by quantum chemical calculations.^2^ Several ML models constructed based on the HTE data of the Suzuki–Miyaura cross-coupling (SMC) reaction have also been reported to optimize the reaction conditions for higher yield.^4^

HTE reaction datasets have been extensively used for the development of ML yield prediction models. A neural network model based on bidirectional encoder representations from transformers (BERT) has been proposed to predict the yields of SMC reactions,^3^ where the chemical language of simplified molecular-input line-entry system (SMILES) was used as the input.^5^ A language-based network architecture as a derivative of the text-to-text transfer transformer (T5)^6^ has been developed for solving multiple tasks in a chemical reaction by one model.^7^ The direct use of chemical graphs as model input has been proposed in combination with graph neural networks.^8^ We have previously proposed a model for yield prediction^9^ composed of a chemical graph-based neural network including a message-passing neural network (MPNN)^10^ and a multihead self-attention technique.^11^ To predict the reaction yield using the network, each compound in a chemical reaction is converted into a chemical graph and input into a MPNN layer. The outputs of the MPNN layer for all of the components are merged by the attention layer. To compensate for a relatively small number of reaction data points (less than 10,000), Mol2Vec^12^ feature vectors are used as the initial node (atom) embeddings, resulting in similar prediction accuracy to RF models using quantum mechanical descriptors^2^ for BHC reaction datasets.

Transformer^11^ architecture was originally proposed in the field of natural language processing, and it has been applied to a variety of fields, including images^13^ and graphs.^14^ The multihead attention mechanism in the transformer can represent the interactions from distinct parts of an input as a network structure, which is important for various targets (e.g., images and graphs). Recently, the transformer architecture has been increasingly used for chemical graphs, leading to higher prediction accuracy than using conventional graph neural networks^10,15^ for molecular property prediction.^16,17^ We expect that the incorporation of the transformer architecture into a chemical graph-based neural network will improve the accuracy of yield prediction.

Here, we propose a chemical graph-based neural network architecture consisting of a MPNN and a transformer, which is called *MPNN-Transformer*. Each chemical graph of the reaction components is converted into a matrix by the MPNN layer, and a set of the matrices is the input of the transformer encoder. In the encoder, the embeddings of the reaction roles, such as the base or solvent, are added to the matrices. The output of the transformer encoder is passed to the multilayer perceptron (MLP) to predict the reaction yield. One advantage of using a transformer encoder is that it can take a variable number of chemical graphs as input and has input order independence. To validate the prediction accuracy of the *MPNN-Transformer* models, we used two widely used HTE datasets, the BHC^2^ and SMC reaction datasets,^18^ and we used a rigorous validation strategy, training and test data splitting, to fairly evaluate the prediction accuracy for chemical reaction components not found in the training dataset. Furthermore, cases in which the prediction accuracies were relatively low for the BHC reaction datasets were investigated based on an outlier relation between the reaction components and their yield in the training dataset.

## 2 Datasets and Methods

### 2.1 Datasets for Yield Prediction

HTE datasets for two types of cross-coupling reactions were prepared to evaluate the performance of the yield prediction models.

#### 2.1.1 BHC Reaction Datasets

The BHC reaction dataset was the Pd-catalyzed BHC reaction dataset reported by Ahneman et al.^2^ containing 3955 reactions as the combinations of 15 aryl halides, 22 additives, four ligands, and three bases. The additives were isoxazoles, which were introduced to evaluate reactions in potentially unfavorable environments. The other reaction conditions, such as the aniline, catalyst, solvent, reaction temperature, reaction time, and reagent amount, were fixed for all of the reactions.

**Figure 1:**
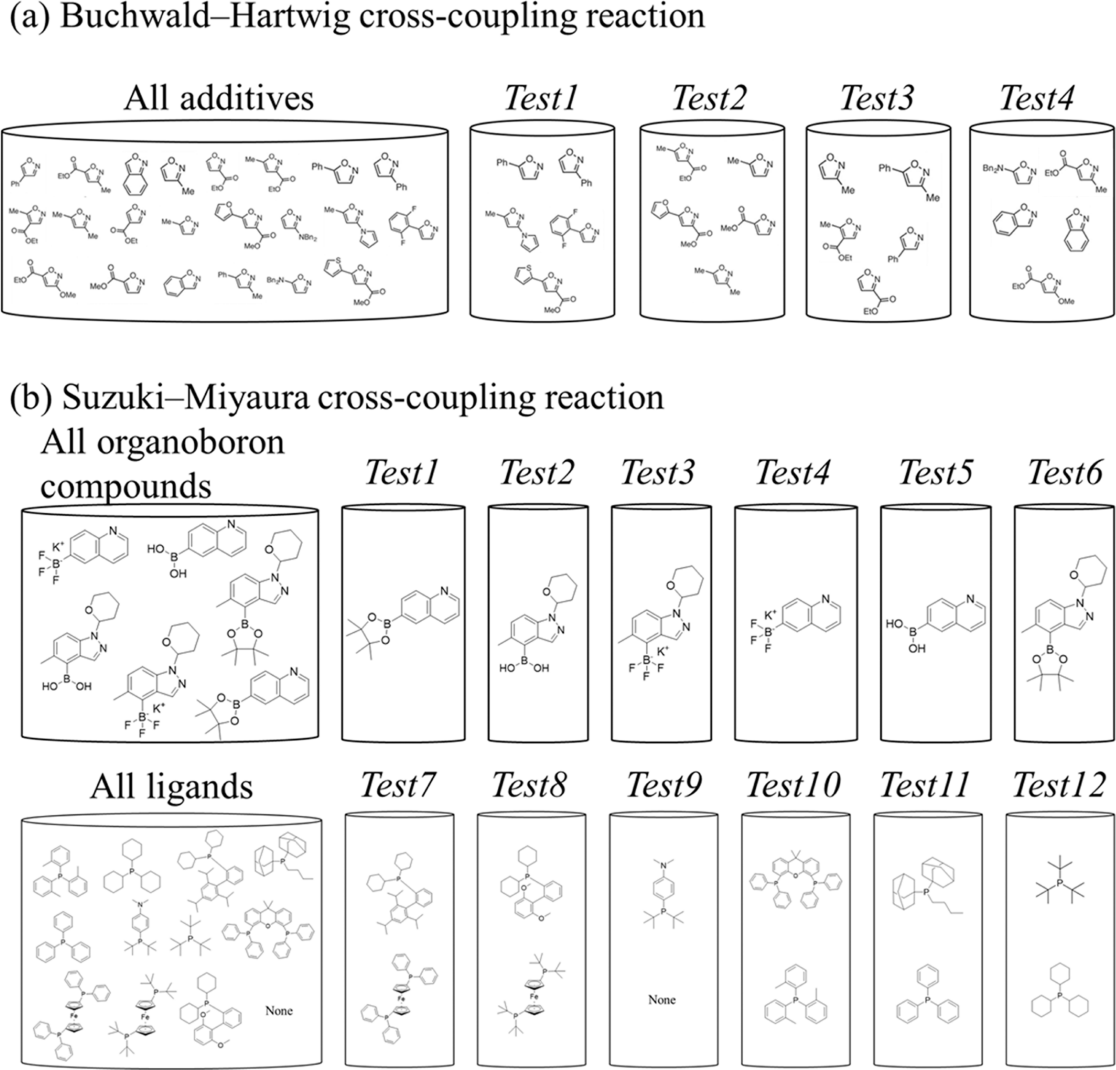
Component-out training and test splitting for the HTE datasets. For the two HTE reaction datasets, BHC reaction dataset (a) and SMC reaction dataset (b), component-out splitting was performed. For (a), the additive was selected as the component, and for (b), the *organoboron* or ligand was selected. The compounds in the cylinder for each dataset exclusively existed in its test dataset.

To evaluate the performance of the yield prediction models, three types of datasets were prepared: *Random*, *Test1–Test4*, and *sTest1–sTest*20 datasets. For the *Random* datasets, the BHC reaction dataset was randomly split into training and test datasets at eight different ratios (70:30, 50:50, 30:70, 20:80, 10:90, 5:95, 2.5:97.5, and 1:99). The splitting was repeated 10 times with different random seeds (a total of 80 datasets). For the ratio of 1:99, each training dataset was restricted to contain the compounds also found in the test dataset, which was the same situation as for the other *Random* datasets. The *Test1–Test4* datasets are defined in the literature,^19^ and they have been used for comparing the model performance in different studies. Each of the four test datasets consisted of all of the reactions involving one of the selected additives (Figure 1a), and the rest of the reactions formed the training set. The same additives were not found in the *Test1–Test4* datasets. The yield distributions of the training and test datasets for the *Test1–Test4* datasets are shown in Figure S1. Moreover, to thoroughly validate the performance of the models for reaction components not found in the training dataset, the *sTest1–sTest*20 datasets were compiled. In this category, each test dataset consisted of one of the four ligands, three out of the 15 aryl halides, and five additives, resulting in 45 reactions per dataset (1 ligand × 3 aryl halides × 5 additives × 3 bases). The five additives in the test datasets were the same as those in the *Test2* dataset. Thus, 20 (4 × (15/3)) distinct test sets were prepared. For each test dataset, the training dataset consisted of the reactions not containing the same ligand, aryl halides, and additives as the test dataset. The total number of reactions for a training dataset was approximately 1830. The three aryl halides for the test datasets were selected in the way so that they had the same scaffold, that is, the functional groups of the three aryl halides were chloro, bromo, and iodo groups.

#### 2.1.2 SMC Reaction Datasets

The SMC reaction dataset was a Pd-catalyzed SMC dataset^18^ consisting of 5760 reaction outcomes as the combinations of seven first reactants, four second reactants, 12 ligands, eight bases, and four solvents. The other reaction conditions, such as the catalyst, reaction temperature, reaction time, and reagent amount, were fixed for all of the reactions. Although the first and second reactants were called *Reactant1* and *Reactant2* in ref (18), we call them *organoboron* and *organohalogen* to focus on the roles of the reactants. It should be noted that the SMC reaction dataset was not combinatorial because for some R groups, the roles of *Reactant1* and *Reactant2* in a reaction were swapped. For the SMC reaction dataset, two types of datasets were prepared: *Random* and *Test1–Test12* datasets. For the *Random* datasets, the SMC reaction dataset was repeatedly randomly split into training and test datasets in eight different ratios (70:30, 50:50, 30:70, 20:80, 10:90, 5:95, 2.5:97.5, and 1:99). The splitting was repeated 10 times with different random seeds (a total of 80 datasets). The *Test1–Test6* datasets corresponded to the six *organoboron* compounds, and each of the six test datasets consisted of all of the reactions including the *organoboron* (Figure 1b, **top**). The *Test7–Test12* datasets were compiled based on the ligands. For each dataset, two of the 12 ligands were randomly selected, and all of the reactions including the selected ligands were included (Figure 1b**, bottom**). For each test dataset, the rest of the reactions were used as training data. The yield distributions for the training and *Test1–Test12* datasets are shown in Figure S2.

### 2.2 ML Models for Comparison

The proposed model was compared with two state-of-the-art models in terms of the prediction accuracy: *Yield-BERT*(3) and *T5Chem*(7) In addition, *XGBoost*(20) using extended connectivity fingerprint with a diameter of 6 (ECFP6)^21^ was also used because of its recently reported high performance for enantioselectivity prediction from the chemical structures of the reaction components.^22^

#### 2.2.1 Yield-BERT

A *Yield-BERT* model is a fine-tuned model of a BERT model.^3^ The BERT model was trained on a large number of SMILES strings of chemical reactions. By fine-tuning the BERT model on a small number of reaction data, the model will learn the relationship between the SMILES strings and yield values. Similar approaches have been previously investigated for the main product prediction^23^ and atom mapping^24^ of chemical reactions. The *Yield-BERT* model was downloaded from the Web site.^25^ Some of the hyper-parameters of the model were tuned before constructing the fine-tuned models (Table S1). To determine the hyper-parameter values, the reactions in a training dataset of *Random* (70:30) of the BHC and SMC reaction datasets were randomly split into training and validation data at a ratio of 9:1. The hyper-parameter values with which the model showed the highest prediction accuracy for the validation data were used to construct the *Yield-BERT* models in this study. Moreover, to determine the batch size of the *Random* datasets, except for *Random* (70:30), the training dataset of each *Random* dataset was randomly split into training and validation data at a ratio of 9:1 (Table S2).

#### 2.2.2 T5Chem

*T5Chem*(7) was developed to solve diverse chemical problems (e.g., retrosynthesis, product prediction, and yield prediction) by one model. *T5Chem* is based on the text-to-text transfer transformer (T5)^6^ architecture, and the SMILES strings in reactions are the inputs of the model. To pretrain the model, 97 million molecules from PubChem^26^ were used. The pretrained *T5Chem* model used in this study was downloaded from the publicly accessible repository.^27,28^ The adjusted hyper-parameters are given in Table S1. These hyper-parameter values were determined in the same way as for *Yield-BERT*. The previously reported performance (*R*^2^) in ref (7) was the square of the correlation coefficient (*R*). For consistency, we recalculated the coefficient of determination (*R*^2^) values for the previously reported datasets (i.e., the *Test1–Test4* datasets in the BHC reaction dataset).

#### 2.2.3 *XGBoost* using ECFP6

A previous study reported that the combination of *XGBoost* and ECFP6 constructed highly predictive models for the enantioselectivity of an S–N acetal formation reaction.^22^ In this study, the *XGBoost* module (version 1.4.0)^20^ was used. For each dataset, the model hyper-parameters were optimized by cross-validation using a training dataset. For the *Random* datasets with a specific training/test ratio, the optimization of the hyper-parameters was performed once using one of the 10 datasets with the same ratio. These optimized hyper-parameters were used for the other models with the same ratio. Early stopping was introduced to reduce the likelihood of overfitting. The tested hyper-parameter values are given in Table S1.

The bit vectors of ECFP6 were constructed with avoiding bit collision.^22^ To produce vectors without bit collision, unique hash values, corresponding to the atomic environment, were collected for all of the compounds in each role of the reactions. The collected hash values were renumbered in decreasing order. Consequently, the sizes of the ECFP6 vectors were different among the roles in the chemical reaction. Because we collected hash vectors for each role, at least one bit could be found for each feature of the ECFP6. To form a reaction descriptor, the component-wise ECFP6 vectors were simply concatenated.

#### 2.2.4 One-hot-RF and Random-RF

As a control, the prediction accuracy of yield prediction models without chemical structural information was investigated. For this purpose, two molecular representations were used in combination with RF regressors: one-hot and random vector representations. A bit in a one-hot vector represents the presence of the compound. Random vector representation simply assigns a random vector to a compound. For the compounds in the BHC reaction dataset, the dimension of the vectors was set to 100, while in the SMC reaction dataset, it was set to 80. These two descriptors represent compound types but not structural information. A reaction descriptor is the concatenation of the one-hot or random vectors for the reaction components. These two representations in combination with RF regressors are called *One-hot-RF* and *Random-RF*. The hyper-parameters of RF were the same as those in ref (29) (n-estimators = 500).

### 2.3 Proposed Yield Prediction Method

#### 2.3.1 Prediction Scheme

**Figure 2:**
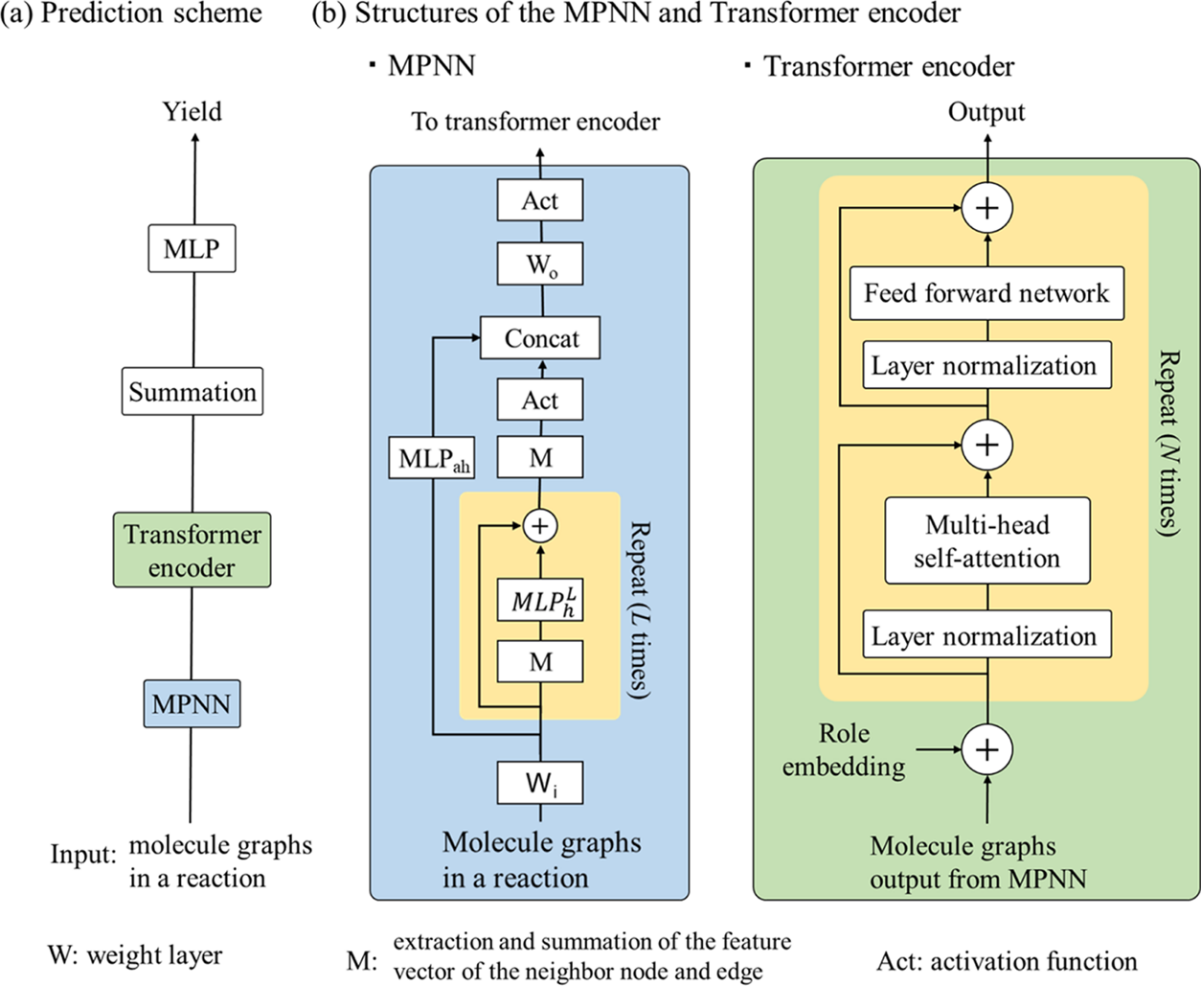
Proposed yield prediction model. (a) Complete scheme for predicting the yield value for a single reaction. (b) Structures of the MPNN and transformer encoder. The yellow boxes are the repetition units. All of the molecules (compounds) in a chemical reaction are processed by the same MPNN. The input form of the transformer encoder is a concatenated matrix of chemical graphs, which is the output of the MPNN.

The proposed neural network architecture is the sequential combination of a MPNN^10^ and a transformer^11^ encoder, which is called *MPNN-Transformer*. An overview of *MPNN-Transformer* is shown in Figure 2a. The output of the MPNN is a set of matrices for the reaction components. These matrices are input into the transformer encoder. The output of the transformer encoder (a matrix) is then vectorized by summation operation, followed by input into a MLP to predict the yield of the reaction. A detailed explanation of the MPNN is shown in Figure 2b. The chemical graphs of the reaction components are the input. For atom embedding of the chemical graphs, Mol2Vec^12^ feature vectors were used, which consider the atomic neighbors up to a radius of 3. The atom feature vectors of the molecules are iteratively updated *L* times by a common message-passing operation. The final output of the MPNN is a set of molecular matrices. These molecular matrices are converted into a new matrix by concatenation. The matrix is then input into the transformer encoder. In the transformer encoder (Figure 2b), role embedding for representing the reaction role for each component is added, such as the reaction product and solvent. Inside the transformer encoder, the combined operation of a multihead self-attention layer and layer normalization is repeated *N* times to represent the interaction among the atoms in the different reaction components. The hyper-parameters of *MPNN-Transformer* are given in Table S3.

### 2.3.2 Pretraining the MPNN by Molecular Contrastive Learning of the Representation via Graph Neural Networks

Molecular contrastive learning of the representation via graph neural networks (MolCLR)^30^ is a self-supervised pretraining framework specifically designed for chemical graphs. Wang et al.^30^ found that MolCLR outperforms previously proposed pretraining methods. In MolCLR, the augmented chemical graphs from an original chemical graph are regarded as positive instances, while the chemical graphs from different molecules are negative instances. By learning the differences between the positive and negative instances, MolCLR can distinguish molecular structures with good generalization. In this study, 3.0 million chemical graphs were prepared for pretraining of the MPNN. All of the compounds in four reaction datasets^2,18,31,32^ and all of the compounds in the Pistachio database (version 2021-10-01) were used. A compound was filtered out if the number of heavy atoms was less than 10 or more than 30, resulting in 3,899,695 compounds. From these compounds, 3.0 million randomly selected compounds were used for pretraining the MPNN. The hyper-parameters of MolCLR were the same as the values reported in,^30^ except for the masking ratio which was fixed to 0.25 in this study. The MPNN was pretrained using MolCLR to attempt to improve the performance of *MPNN-Transformer*. In contrast, the transformer encoder and final MLP were not pretrained. The number of pretrained compounds is shown in parentheses after the model name. For example, *MPNN-Transformer(3.0m)* is the *MPNN-Transformer* model trained on 3.0 million compounds.

#### 2.3.3 Chemical Graph Augmentation for MolCLR

**Figure 3:**
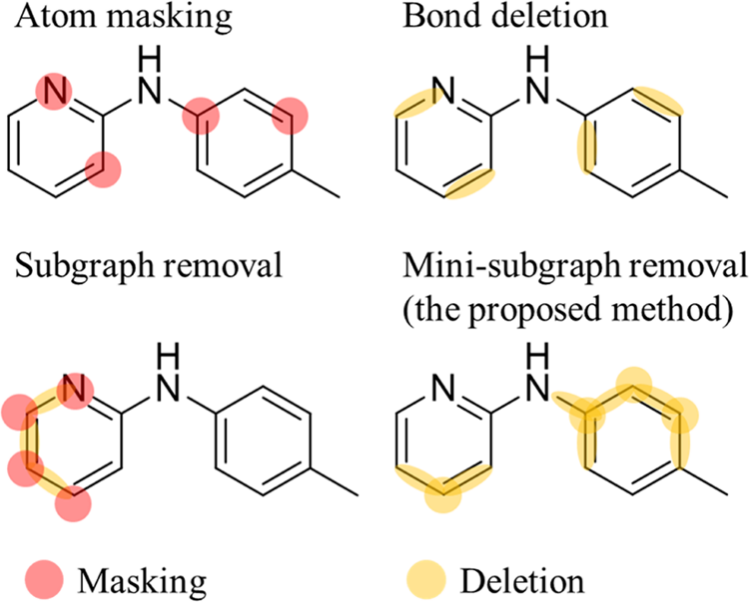
Augmentation in MolCLR. Atom masking, bond deletion, and subgraph removal are previously proposed methods for augmentation in the original paper.^30^ Mini-subgraph removal is proposed in this study.

We propose a different chemical graph augmentation strategy for MolCLR from the ones reported in ref (30). In the original study of MolCLR, atom masking, bond deletion, and subgraph removal were tested as augmentation methods, and subgraph removal was found to be the best. For subgraph removal, if a large subgraph is masked, the remaining atoms might not contain masked atom information in their atom vectors. However, because our atom embedding is Mol2Vec with a radius of 3, to keep the information on the deleted parts of a chemical graph in the remaining atoms, we delete small parts of a chemical graph, which is called mini-subgraph removal. For mini-subgraph removal, a center atom is randomly selected, and neighbor atoms up to radius 1 are deleted (Figure 3). This operation is repeated until the number of deleted atoms reaches a predetermined number (in this study, 0.25 of the entire chemical graph size).

#### 2.3.4 Ensemble Approach

A simple ensemble approach was also tested: averaging the outputs of multiple ML models with different random seeds. This approach is effective for both traditional ML and deep learning models.^33^ In this study, for each prediction with a ML architecture, 50 models were created with different random seeds. By randomly selecting 10 models from the pool of 50 models, five ensemble models were created, except for the *Random* datasets of the two HTE datasets. For the *Random* datasets, one ensemble model was used. The yield predicted by each ensemble model is the average of the outputs of 10 models. The same seed values were used for all of the models. This ensemble approach was applied to all of the ML modeling methods in this study, and the reported values of the model performance were calculated based on the output values of the ensemble approach.

### 2.4 Evaluation Metric

To represent the prediction accuracy of a regression model, the coefficient of determination (*R*^2^), root mean squared error, and mean absolute error are commonly used. Based on the idea that a model showing significant prediction errors even for a few reactions is unreliable, the root mean squared error and *R*^2^ are appropriate. Furthermore, to easily understand the statistical goodness of fit, *R*^2^, which has a range of [0, 1] when the sum of the prediction square errors is smaller than that of the squares of the test data, was selected as the evaluation metric.

## 3 Results and Discussion

### 3.1 Study Design

**Figure 4:**
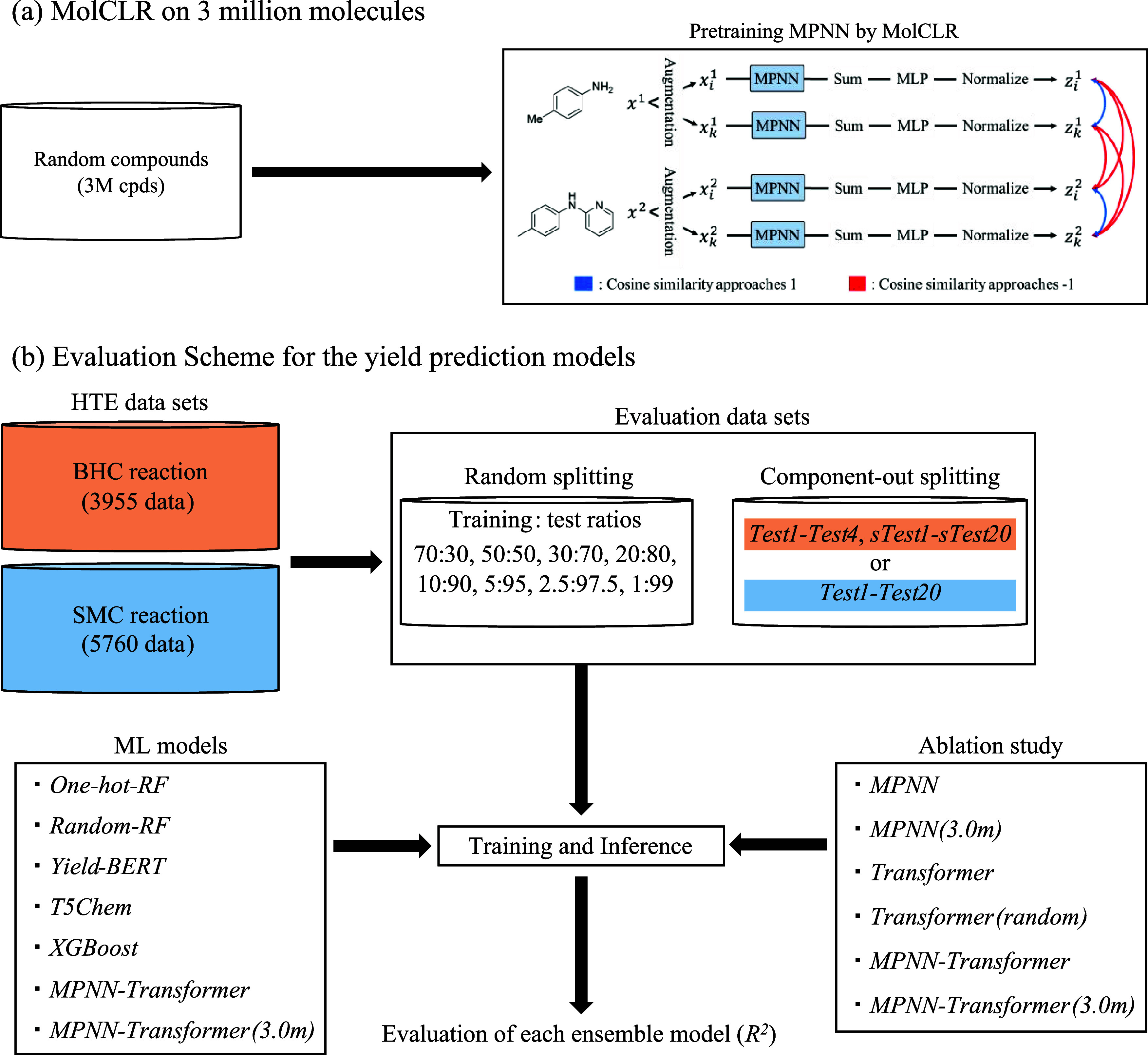
Study overview. (a) MolCLR pretraining. Randomly selected compounds were used for the pretraining. (b) Evaluation workflow. Evaluation datasets were prepared from the HTE datasets by random and component-out splitting. ML yield prediction models were built using the training datasets and evaluated for the test datasets in *R*^2^. The ensemble approach was applied to all methods, and the ablation study for *MPNN-Transformer* was conducted.

The yield prediction models introduced in **2**. **Datasets and Methods** were evaluated using two HTE reaction datasets with several data-splitting strategies. Figure 4 presents an overview of this study. For the proposed *MPNN-Transformer*, the MPNN was pretrained with MolCLR using a set of 3 million compounds (Figure 4a). For the evaluation, random and component-out BHC and SMC HTE datasets were prepared, and the yield prediction models were evaluated based on the *R*^2^ values for the test datasets (Figure 4b). The performance of *MPNN-Transformer* was investigated with or without the MolCLR pretraining. The ensemble approach was introduced to all methods, and the ablation study was conducted for *MPNN-Transformer* to understand the importance of its component (Figure 4b).

### 3.2 Prediction for the BHC Reaction Datasets

#### 3.2.1 Prediction Accuracy for the *Random* BHC Reaction Datasets

The prediction accuracies for the *Random* datasets of the BHC reaction are given in Tables 1 and S4. For *MPNN-Transformer*, the prediction accuracies with and without MolCLR-based pretraining are also given (*MPNN-Transformer(3.0m)*). All of the ML models achieved high prediction accuracy values when they were trained on a large number of reactions. For the *Random* datasets, because compounds were shared between the training and test datasets, the prediction task was relatively easy. Although the prediction accuracy values for *One-hot-RF* and *Random-RF* were reasonable when a large training dataset was used (e.g., 70:30 and 50:50), they were always inferior to those of the other models using chemical structural information as descriptors. As expected, the prediction accuracy decreased as the training dataset size decreased. However, when all of the tested models were trained on 197 reactions (5:95), the average *R*^2^ values reached 0.7 (excluding the *DFT-RF*, Table 1). Moreover, when the tested models were trained on 40 random reactions (1:99), *T5Chem*, *Yield-BERT*, and *XGBoost* achieved average *R*^2^ values of greater than 0.4. However, *MPNN-Transformer(3.0m)* showed poor performance for the same very small training dataset (*R*^2^ = 0.21).

**Table 1:** Prediction Accuracies (*R*^2^) for the *Random* Datasets of the BHC Reaction^a^

training:test	70:30	50:50	30:70	20:80	10:90	5:95	2.5:97.5	1:99
*one-hot-RF*	0.89 (0.01)	0.87 (0.01)	0.84 (0.01)	0.81 (0.01)	0.74 (0.02)	0.64 (0.04)	0.49 (0.09)	0.25 (0.11)
*random-RF*	0.92 (0.01)	0.90 (0.01)	0.86 (0.01)	0.83 (0.01)	0.76 (0.02)	0.65 (0.04)	0.53 (0.03)	0.27 (0.10)
*DFT-RF	0.92	0.90	0.85	0.81	0.77	0.68	0.59	
*yield-BERT*	0.97 (0.00)	0.93 (0.01)	0.88 (0.01)	0.86 (0.01)	0.81 (0.01)	0.73 (0.02)	0.55 (0.06)	0.43 (0.15)
*T5Chem*	0.97 (0.00)	0.96 (0.00)	0.93 (0.01)	0.90 (0.01)	0.83 (0.02)	0.76 (0.01)	0.65 (0.03)	0.42 (0.11)
*XGBoost*	0.95 (0.00)	0.94 (0.00)	0.90 (0.01)	0.88 (0.01)	0.82 (0.01)	0.76 (0.02)	0.65 (0.03)	0.45 (0.10)
*MPNN-transformer*	0.97 (0.00)	0.96 (0.00)	0.93 (0.01)	0.90 (0.01)	0.83 (0.02)	0.75 (0.02)	0.63 (0.04)	0.40 (0.10)
*MPNN-transformer(3.0m)*	0.97 (0.00)	0.97 (0.00)	0.94 (0.01)	0.91 (0.01)	0.84 (0.01)	0.77 (0.02)	0.64 (0.05)	0.21 (0.09)

a For each model and dataset, the average (standard deviation) of the *R*^2^ value for the *Random* datasets of the BHC reaction using the five ensemble models is reported. *For *DFT-RF*,^2^ the reported values are given, so direct comparison may not be appropriate. For each test dataset, the highest *R*^2^ values are highlighted in bold.

#### 3.2.2 Prediction Accuracy for the Additive-Out BHC Reaction Datasets

The prediction accuracies for the *Test1–Test4* datasets are given in Tables 2 and S5. The data splitting was based on the scheme in Figure 1. For these extrapolation-oriented datasets in terms of additives, *MPNN-Transformer(3.0m)* showed overall high prediction performance. Introducing MolCLR into the *MPNN-Transformer* model contributed to the performance improvement for the *Test3* dataset. In addition, compared with the other models, *MPNN-Transformer* and *MPNN-Transformer(3.0m)* were able to predict the yields of the *Test4* dataset with high accuracy. The *One-hot RF* and *Random-RF* models showed reasonable prediction accuracy, but overall their prediction accuracies were lower than those of the other models, meaning that chemical structural information is important to predict the yields for these extrapolation-oriented datasets. When the ensemble approach was introduced, the prediction accuracy improved for the neural network-based models; however, *XGBoost* showed little change in the accuracy.

**Table 2:** Prediction Accuracies (*R*^2^) for the *Test1–Test4* Datasets of the BHC Reaction^a^

	*Test1*	*Test2*	*Test3*	*Test4*
*one-hot-RF*	0.69 (0.00)	0.67 (0.00)	0.50 (0.00)	0.48 (0.00)
*random-RF*	0.69 (0.00)	0.82 (0.00)	0.52 (0.00)	0.42 (0.00)
*yield-BERT*	0.84 (0.01)	0.83 (0.01)	0.74 (0.01)	0.49 (0.02)
*T5Chem*	0.82 (0.01)	0.91 (0.01)	0.76 (0.01)	0.55 (0.01)
*XGBoost*	0.88 (0.00)	0.89 (0.00)	0.60 (0.00)	0.58 (0.00)
*MPNN-transformer*	0.87 (0.01)	0.88 (0.01)	0.59 (0.03)	0.64 (0.01)
*MPNN-transformer(3.0m)*	0.87 (0.01)	0.90 (0.00)	0.74 (0.01)	0.61 (0.01)
*DFT-RF(2)	0.80	0.77	0.64	0.54
*Mol2Vec-MPNN(9)	0.92	0.88	0.60	0.39

a For each model and dataset, the average (standard deviation) of the *R*^2^ value for the *Test1–Test4* datasets of the BHC reaction using the five ensemble models is reported. *For *DFT-RF*(2) and *Mol2Vec-MPNN*,^9^ the reported values are given, so direct comparison may not be appropriate. For each test dataset, the three highest *R*^2^ values are highlighted in bold.

#### 3.2.3 Difficulty in Yield Prediction for the *Test3* and *Test4* Datasets

**Figure 5:**
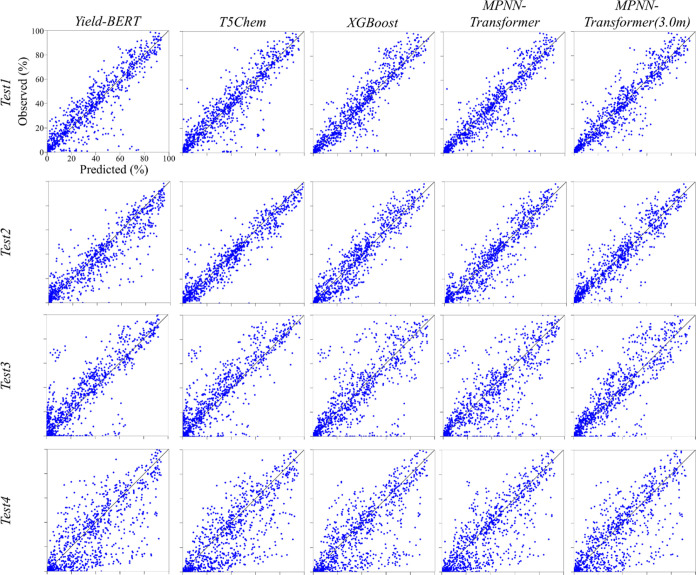
Observed yield versus predicted yield plots for the *Test1–Test4* datasets of the BHC reaction. The observed yield versus predicted yield plots are shown for the exhaustive combinations of the ML models and test datasets.

**Figure 6:**
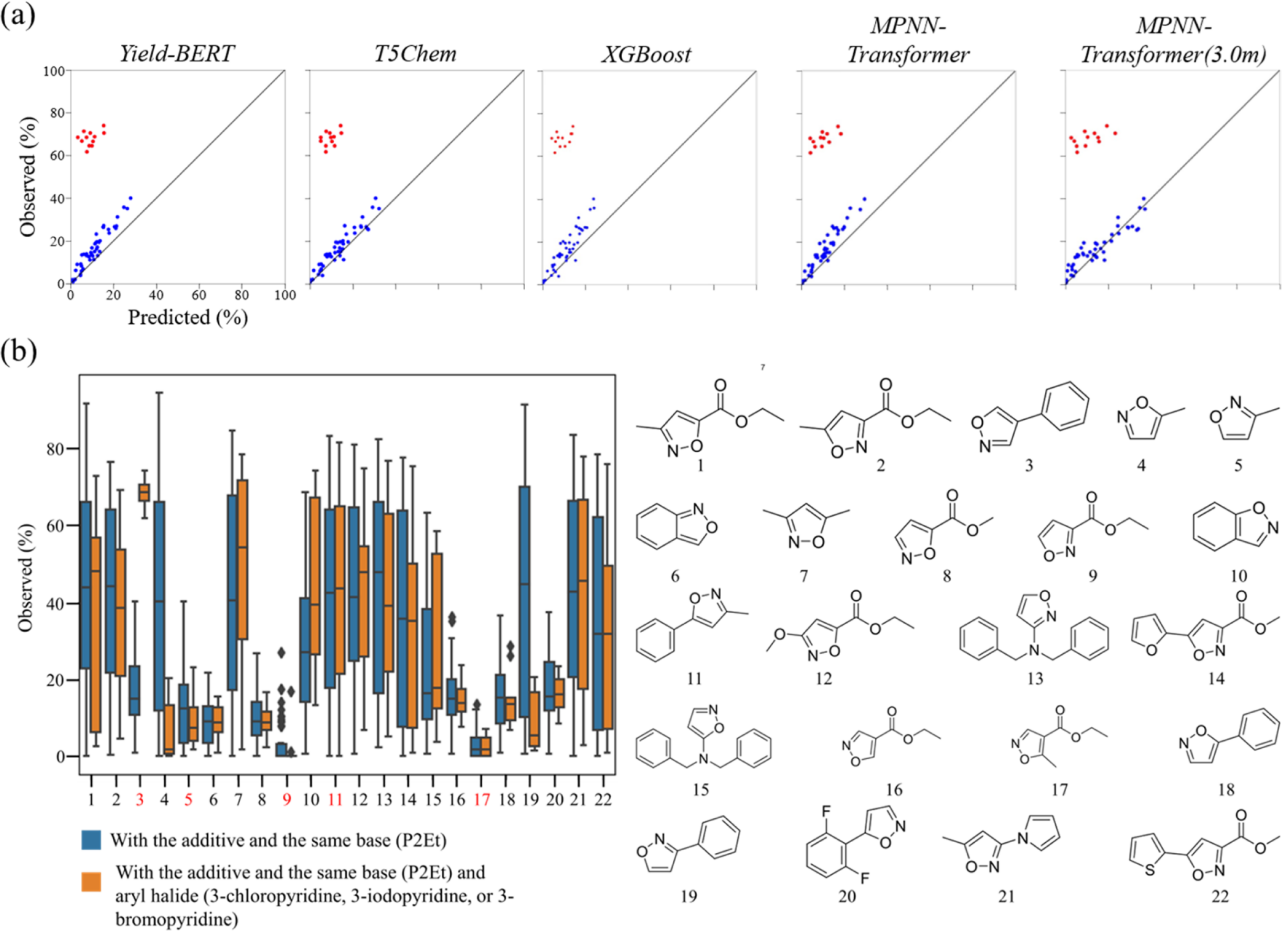
Outlier analysis for the BHC reaction. (a) Observed yield versus predicted yield plots for the combination of the 4-phenylisoxazole additive and P2Et base. The red points represent the reactions containing one of the three aryl halides 3-chloropyridine, 3-iodopyridine, or 3-bromopyridine. (b) Additive-wise box plots of the observed yields for the reactions containing P2Et (left) and the structural formulas of the additives (right). The blue box plots are for the reactions without the specific aryl halides, while the orange boxes are for the reactions with one of the three aryl halides in (a). The red numbers on the *x* axis indicate the five additives in the test data of the *Test3* dataset.

Plots of the observed yield versus predicted yield for the *Test1–Test4* datasets are shown in Figure 5, which are categorized in terms of the prediction model. For the *Test3* and *Test4* datasets (the bottom two rows in Figure 5), there was a similar pattern of the outliers (apart from the diagonal line) irrespective of the prediction models. The extracted observed yield versus predicted yield plots for the test reactions including the 4-phenylisoxazole additive and P2Et base are shown in Figure 6a. The outlier reactions in the yield prediction in Figure 6a (red circles) were reactions containing the same additive, same base, and one of the three aryl halides 3-chloropyridine, 3-iodopyridine, or 3-bromopyridine. These outlier reactions were common to all of the models. Additive-wise box plots of the observed yields for the reactions containing the same base (P2Et) are shown in Figure 6b. In Figure 6b, the blue boxes are for the reactions without the three aryl halides, while the orange boxes are for the reactions containing one of the three aryl halides. Except for 4-phenylisoxazole (number 3 in Figure 6b), the blue and orange box plots of the four additives in the test data of the *Test3* dataset (numbers 5, 9, 11, and 17 in Figure 6b) showed similar trends to the other additives of the training data for the *Test3* dataset. However, for 4-phenylisoxazole, the observed yield values for the three aryl halides were much higher than those for the rest of the aryl halides. Although it is interesting to interpret this phenomenon from a chemical reaction point of view, accurate yield prediction for such data points generally seems to be difficult. For the *Test4* dataset, an outlier pattern was also observed. The yield prediction for the reactions including benzo[c]isoxazole as an additive always showed very low accuracy irrespective of the model (Figure S3).

#### 3.2.4 Prediction Accuracy for the *sTest1–sTest20* Datasets of the BHC Reaction

A further rigorous validation of the prediction ability of the ML models for the reactions containing reaction components not found in the training dataset was performed. The prediction accuracies (*R*^2^) for all of the datasets (*sTest1*–*sTest20*) are given in Tables S6 and S7. As expected, *One-hot-RF* and *Random-RF* did not work at all, and their performance was even significantly poorer than for the *Test1–Test4* datasets (Table 2), suggesting that *sTest1*–*sTest20* were eligible datasets for measuring the extrapolation ability of the models. Overall, the *MPNN-Transformer* models worked the best, followed by *XGBoost*. By counting the number of test datasets where a model was ranked within the top three models and the *R*^2^ value was greater than 0 in Table S7, *MPNN-Transformer(3.0m)* was selected 11 times out of the 20 test trials, while *XGBoost* was selected nine times and *T5Chem* was selected six times. *MPNN-Transformer(3.0m)* was overall better for predicting the yield of the chemical reactions containing multiple components not found in the training dataset although the suitable ML models highly depended on the test dataset. Furthermore, pairwise comparison between *MPNN-Transformer* and *MPNN-Transformer(3.0m)* showed that *MPNN-Transformer(3.0m)* worked well in 7 out of the 12 datasets, which the *R*^2^ values were greater than 0 and differed between the two models. The contrastive learning technique contributed to the performance improvement of the *MPNN-Transformer* model.

### 3.3 Prediction for the SMC Reaction Datasets

#### 3.3.1 Prediction Accuracy for the *Random* SMC Reaction Datasets

The prediction accuracies of the models for the *Random* datasets of the SMC reaction are given in Tables 3 and S8. All of the models except for *Yield-BERT* exhibited high accuracy when the training dataset size was large. The same explanation as that for the BHC reaction could be valid for the high predictive ability of the models: compound overlap between the training and test reactions. Even the representations not using chemical structural information, *One-hot-RF* and *Random-RF*, achieved reasonable prediction accuracy, suggesting chemical structural information only slightly contributed to the prediction accuracy. When the training dataset size was 58 (1:99 in Table 3), the average *R*^2^ value for *XGBoost* decreased to 0.32. The accuracies of the proposed *MPNN-Transformer* models decreased when the training dataset size was 58, and they showed poorer predictive ability than *One-hot-RF* and *Random-RF*. This could be because of the insufficient generalization ability of neural network models when the training dataset is too small to train a large number of parameters. The MPNN part of the *MPNN-Transformer* model was pretrained, while the rest of the network was not. Differing from the prediction trials for the BHC reaction datasets, the prediction accuracies of the *MPNN-Transformer* models for this sized training dataset were relatively high, meaning that the models fitted to the training data relatively well.

**Table 3:** Prediction Accuracies (*R*^2^) for the *Random* Datasets of the SMC Reaction^a^

training:test	70:30	50:50	30:70	20:80	10:90	5:95	2.5:97.5	1:99
*one-hot-RF*	0.84 (0.01)	0.82 (0.00)	0.78 (0.01)	0.75 (0.01)	0.68 (0.01)	0.61 (0.03)	0.51 (0.04)	0.24 (0.08)
*random-RF*	0.84 (0.01)	0.82 (0.01)	0.78 (0.00)	0.75 (0.01)	0.68 (0.01)	0.60 (0.01)	0.46 (0.06)	0.27 (0.05)
*yield-BERT*	0.80 (0.01)	0.78 (0.06)	0.73 (0.01)	0.68 (0.01)	0.58 (0.02)	0.49 (0.02)	0.36 (0.06)	0.25 (0.04)
*T5Chem*	0.88 (0.01)	0.87 (0.01)	0.82 (0.01)	0.79 (0.01)	0.69 (0.01)	0.58 (0.03)	0.41 (0.05)	0.20 (0.11)
*XGBoost*	0.86 (0.01)	0.85 (0.00)	0.81 (0.00)	0.77 (0.01)	0.70 (0.01)	0.63 (0.01)	0.51 (0.04)	0.32 (0.07)
*MPNN-transformer*	0.89 (0.01)	0.86 (0.01)	0.82 (0.01)	0.78 (0.01)	0.67 (0.02)	0.56 (0.02)	0.41 (0.02)	0.09 (0.14)
*MPNN-transformer(3.0m)*	0.88 (0.01)	0.85 (0.01)	0.79 (0.01)	0.75 (0.01)	0.69 (0.02)	0.59 (0.01)	0.44 (0.03)	0.09 (0.14)

a For each model and dataset, the average (standard deviation) of the *R*^2^ value for the *Random* datasets of the SMC reaction using the five ensemble models is reported. For each test dataset, the highest *R*^2^ value is highlighted in bold.

The prediction accuracies for the *Random* datasets of both the BHC and SMC reactions revealed that the *T5Chem* and the *MPNN-Transformer* models were better when the number of training data was large, while *XGBoost* was better when the number of training data was small.

#### 3.3.2 Prediction Accuracy for the Component-Out SMC Reaction Datasets

Observed yield versus predicted yield plots for the *Test1*–*Test12* datasets of the SMC reaction are shown in Figure S4. For the *Test1*, *Test3*, *Test4*, and *Test5* datasets, all of the ML models failed to predict the reaction yield, the *R*^2^ values were relatively low, and *Random-RF* showed the best performance (Tables S9 and S10). *MPNN-Transformer(3.0m*) seemed to be overfitted to the training data for these datasets, as shown in Figure S5. These datasets were created by organoboron-based splitting. The training datasets for the *Test1–Test6* datasets contained only two organoboron compounds, and the rest of the reaction components were the same because the organoboron compounds in the SMC dataset consisted of two scaffolds, unlike typical HTE datasets.^18^ Therefore, for these datasets, construction of a highly predictive yield prediction model was difficult.

The prediction accuracies for the *Test2*, *Test6*, and *Test7–Test12* datasets are given in Tables 4 and S10, focusing only on the test datasets for which *R*^2^ > 0. *MPNN-Transformer(3.0m)* and *XGBoost* worked better than the other models. Comparing these two models, *MPNN-Transformer(3.0m)* worked better than *XGBoost* for five out of seven datasets based on Welch’s *t* test analysis of *R*^2^ (Table S11). Moreover, there were very large differences in the accuracies for some of the datasets. For the *Test6* and *Test7* datasets, *MPNN-Transformer(3.0m)* achieved *R*^2^ = 0.73 and 0.60, while *XGBoost* achieved *R*^2^ = 0.08 and 0.39. However, for the *Test2* dataset, these two models did not work (*R*^2^ = −0.04 for *MPNN-Transformer(3.0m)*), while *Yield-BERT* and *T5Chem* showed relatively high prediction accuracies (*R*^2^ = 0.48 for *Yield-BERT* and *R*^2^ = 0.63 for *T5Chem*).

**Table 4:** Prediction Accuracies (*R*^2^) for the *Test2*, *Test6*, and *Test7–Test12* Datasets of the SMC Reaction^a^

	*Test2*	*Test6*	*Test7*	*Test8*	*Test9*	*Test10*	*Test11*	*Test12*
*one-hot-RF*	–0.43 (0.00)	–0.37 (0.00)	0.00 (0.00)	0.37 (0.00)	0.51 (0.00)	–0.21 (0.00)	0.57 (0.00)	0.64 (0.00)
*random-RF*	–0.01 (0.01)	0.27 (0.01)	0.22 (0.00)	0.14 (0.00)	0.40 (0.00)	0.04 (0.01)	0.28 (0.00)	0.43 (0.00)
*yield-BERT*	0.48 (0.02)	0.64 (0.00)	–0.50 (0.06)	0.35 (0.02)	0.06 (0.04)	–0.39 (0.08)	0.24 (0.02)	0.61 (0.00)
*T5Chem*	0.63 (0.02)	0.71 (0.01)	–0.02 (0.07)	0.28 (0.01)	0.31 (0.04)	–0.59 (0.04)	0.26 (0.04)	0.66 (0.02)
*XGBoost*	–0.53 (0.00)	0.08 (0.00)	0.39 (0.00)	0.59 (0.00)	0.66 (0.00)	0.30 (0.00)	0.32 (0.01)	0.66 (0.00)
*MPNN-transformer*	–0.54 (0.01)	0.72 (0.01)	0.50 (0.02)	0.56 (0.02)	0.39 (0.01)	–0.45 (0.05)	0.53 (0.02)	0.66 (0.00)
*MPNN-transformer(3.0m)*	–0.04 (0.06)	0.73 (0.02)	0.60 (0.01)	0.68 (0.01)	0.38 (0.02)	–0.03 (0.01)	0.59 (0.01)	0.68 (0.01)

a For each model and dataset, the average (standard deviation) of the *R*^2^ value for the *Test2*, *Test6*, *Test7–Test12* datasets of the SMC reaction using the five ensemble models is reported. For each test dataset, the three highest *R*^2^ values are highlighted in bold, unless *R*^2^ < 0.

#### 3.3.3 Performance Differences for the *Test2* and *Test6* Datasets of the SMC Reaction

**Figure 7:**
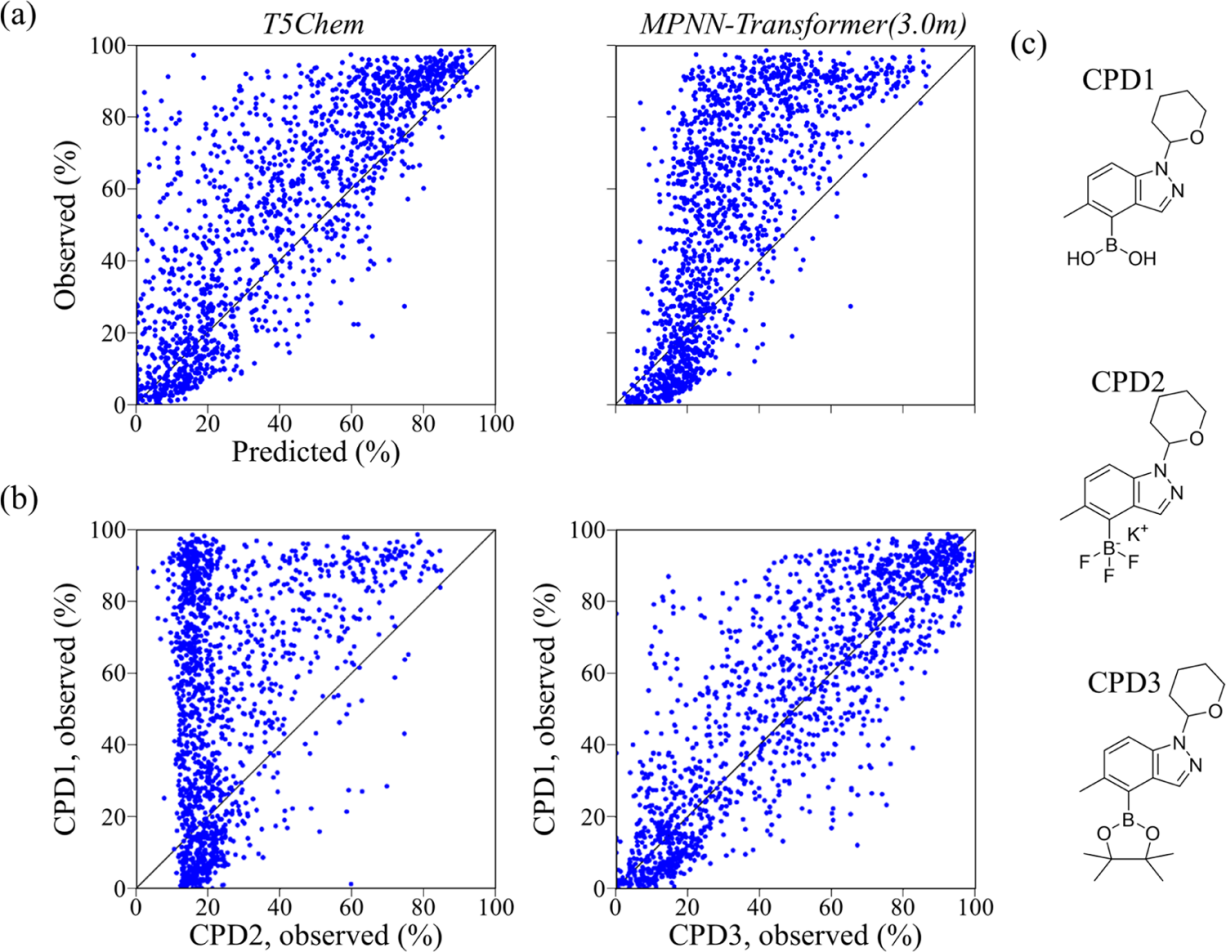
Comparison of the prediction abilities of *T5Chem* and *MPNN-Transformer(3.0m)* for the *Test2* dataset. (a) Observed yield versus predicted yield plots for the *Test2* dataset using *T5Chem* and *MPNN-Transformer(3.0m)*. In the *Test2* dataset, CPD1 was in the test set, and CPD2 and CPD3 were in the training set. (b) Observed yield versus predicted yield plots for the same reaction components except that the two *organoboron* components are shown as scatterplots. The *y* axis of the two plots is the observed yield for the reaction containing CPD1, while the *x* axis is for the reaction containing CPD2 and CPD3. (**c**) Structural formulas of CPD1–CPD3.

For the *Test2* dataset, the *Yield-BERT* and *T5Chem* models worked better than the other models. These natural language-based neural network models use reaction SMILES as the input for the yield prediction. To investigate possible reasons for the performance difference between the language-based and chemical graph-based models, the prediction results of *T5Chem* and *MPNN-Transformer(3.0m)* were compared. The plots of the observed yield versus predicted yield for the *Test2* dataset by *T5Chem* (*R*^2^ = 0.63) and *MPNN-Transformer(3.0m)* (*R*^2^ = −0.04) are shown in Figure 7a. The observed yield versus predicted yield plots for the reactions consisting of the same reaction components except for the *organoboron* compounds are shown in Figure 7b. The three *organoboron* compounds contained in the *Test2* and *Test6* datasets are represented by CPD1–CPD3, as shown in Figure 7c. The *Test2* dataset consisted of the reactions including CPD1, and the *Test6* dataset included CPD3. The yields of the reactions containing CPD1 were plotted (*y* axis) against those containing CPD2 (*x* axis) (Figure 7b, left). CPD1 and CPD3 have similar structures (Figure 7b, right), and the correlation coefficient of the observed yields for these two datasets was 0.86. Conversely, the yields for the reactions containing CPD1 and CPD2 showed little correlation (*R*^2^ = 0.36). High correlation was observed between the values predicted by *T5Chem* and the observed yields for the CPD3 dataset (*R*^2^ = 0.96, Figure S6). Thus, *T5Chem* seemed to predict the yields based on the information on the reactions containing CPD3, which had a high correlation coefficient with the yields of the reactions containing CPD1, leading to *T5Chem* showing high prediction accuracy. The canonical SMILES representations of the three compounds are

CPD1: Cc1ccc2c(cnn2C2CCCCO2)c1B(O)O

CPD2: Cc1ccc2c(cnn2C2CCCCO2)c1[B-](F)(F)F.[K+]

CPD3: Cc1ccc2c(cnn2C2CCCCO2)c1B1OC(C)(C)C(C)(C)O1

The boron atom with a negative formal charge seemed to be recognized as being distinct from the boron atom without a formal charge. The high prediction accuracy of *T5Chem* for the *Test6* dataset can be rationalized in the same way.

### 3.4 Ablation Study

The proposed neural network structure consists of a MPNN and a transformer encoder, and improvement of the performance was observed when the network was pretrained by contrastive learning. An ablation study, removing a component of the network, was performed by measuring the prediction accuracy for the additive-out BHC reaction datasets and component-out SMC datasets. In the following subsections, *MPNN* refers to the model architecture consisting of the MPNN part of *MPNN-Transformer* in Figure 2, *MPNN(3.0m)* refers to the MPNN model pretrained using 3.0 million compounds, and *Transformer* refers to the model architecture consisting of the transformer encoder part. *Transformer(random)* refers to the model architecture consisting of the transformer encoder whose input is a set of randomly created atomic feature vectors.

#### 3.4.1 Ablation Study Using the Additive-Out BHC Reaction Datasets

The prediction accuracies of *MPNN*, *MPNN(3.0m)*, *Transformer*, *Transformer(random)*, *MPNN-Transformer*, and *MPNN-Transformer(3.0m)* for the *Test1–Test4* datasets of the BHC reaction are given in Table 5, and the individual model prediction results are given in Table S12. *Transformer(random)* showed low overall prediction accuracy, indicating that the Mol2Vec atom vectors contributed to the yield prediction for the BHC reaction dataset. For the *Test1* and *Test2* datasets, all of the models except for *Transformer(random)* showed high prediction accuracy. For the *Test3* dataset, although *Transformer* and *MPNN-Transformer* were less accurate than the other models, *MPNN-Transformer(3.0m)* and *MPNN(3.0m)* were more accurate. For the *Test4* dataset, *MPNN-Transformer* was clearly superior to *Transformer*. There was little difference in the prediction accuracies between *MPNN(3.0m)* and *MPNN-Transformer(3.0m)* for these datasets.

**Table 5:** Prediction Accuracies (*R*^2^) in the Additive-Out Ablation Study for the *Test1–Test4* Datasets of the BHC Reaction^a^

	*Test1*	*Test2*	*Test3*	*Test4*
*MPNN*	0.93 (0.00)	0.90 (0.00)	0.71 (0.00)	0.52 (0.00)
*MPNN(3.0m)*	0.87 (0.00)	0.88 (0.00)	0.74 (0.00)	0.62 (0.00)
*transformer*	0.91 (0.00)	0.91 (0.00)	0.64 (0.01)	0.44 (0.00)
*transformer(random)*	0.56 (0.00)	0.61 (0.00)	0.21 (0.00)	0.29 (0.00)
*MPNN-transformer*	0.87 (0.01)	0.88 (0.01)	0.59 (0.03)	0.64 (0.01)
*MPNN-transformer(3.0m)*	0.87 (0.01)	0.90 (0.00)	0.74 (0.01)	0.61 (0.01)

a For each model and dataset, the average (standard deviation) of the *R*^2^ value for the *Test1–Test4* datasets of the BHC reaction using the five ensemble models is reported. *Transformer:* the transformer encoder layer. *Transformer(random)*: the transformer encoder layer with random input vectors. *MPNN*: the MPNN part of the proposed model in Figure 2. *MPNN(3.0m)*: MPNN with contrastive learning using 3.0 million compounds. For each test dataset, the highest *R*^2^ value is highlighted in bold.

#### 3.4.2 Ablation Study Using the Component-Out SMC Reaction Datasets

The same ablation study as that for the BHC reaction datasets was performed using the SMC component-out datasets (Table 6). The prediction accuracies for the *Test1–Test6* datasets are given in Table S13, and the individual model prediction results are given in Table S14. For most of the datasets, the prediction accuracy by *Transformer(random)* was lower than that by *MPNN-Transformer(3.0m)*. However, to our surprise, for the *Test9* dataset, randomly assigned molecular vectors worked better than MPNN feature vectors in combination with the transformer encoder. Because the *Test9* dataset contained “none” (no ligand), the MPNN output for this ligand was the zero vector. Focusing only on the test datasets for which *R*^2^ > 0, contrastive learning contributed to the performance improvement for both *MPNN* and *MPNN-Transformer* (based on Welch’s *t* test analysis of *R*^2^, Tables S15 and S16). For all of the datasets, *Transformer* showed higher prediction accuracy than *MPNN* and *MPNN(3.0m)*. Overall, *MPNN-Transformer(3.0m)* showed the best performance for the most datasets. From these ablation studies using BHC and SMC datasets, *MPNN-Transformer(3.0m)* showed the most stable and best performance, followed by *MPNN(3.0m)*.

**Table 6:** Prediction Accuracies (*R*^2^) in the Component-Out Ablation Study for the *Test7–Test12* Datasets of the SMC Reaction^a^

	*Test7*	*Test8*	*Test9*	*Test10*	*Test11*	*Test12*
*MPNN*	0.30 (0.00)	0.46 (0.00)	0.34 (0.00)	–0.91 (0.00)	0.12 (0.01)	0.55 (0.00)
*MPNN(3.0m)*	0.49 (0.00)	0.58 (0.00)	0.34 (0.00)	–0.55 (0.01)	0.19 (0.00)	0.46 (0.00)
*transformer*	0.51 (0.00)	0.62 (0.00)	0.41 (0.01)	0.09 (0.01)	0.55 (0.01)	0.62 (0.01)
*transformer (random)*	0.03 (0.01)	0.05 (0.01)	0.60 (0.01)	–0.42 (0.02)	0.55 (0.00)	0.40 (0.01)
*MPNN-transformer*	0.50 (0.02)	0.56 (0.02)	0.39 (0.01)	–0.45 (0.05)	0.53 (0.02)	0.66 (0.00)
*MPNN-transformer(3.0m)*	0.60 (0.01)	0.68 (0.01)	0.38 (0.02)	–0.03 (0.01)	0.59 (0.01)	0.68 (0.01)

a For each model and dataset, the average (standard deviation) of the *R*^2^ value for the *Test7–Test12* datasets of the SMC reaction using the five ensemble models is reported. For each test dataset, the highest *R*^2^ value is highlighted in bold.

## 4 Conclusions

The development of models for reaction yield prediction is important in chemoinformatics and organic chemistry. Here, we propose a *MPNN-Transformer* architecture to predict the reaction yields for HTE datasets. As a novel methodological point, a MPNN and a transformer encoder with reaction role embeddings are sequentially connected, with which a variable number of reaction components can be handled. From rigorous validation using two HTE reaction datasets, the proposed *MPNN-Transformer* architecture with molecular contrastive learning achieved the highest overall prediction ability among state-of-the-art data-driven yield prediction models. For the reaction datasets consisting of extrapolated reaction components, the contrastive learning technique contributed to the higher prediction accuracy of *MPNN-Transformer*.

From control calculations using a random vector representation, it was determined to be difficult to construct meaningful yield prediction models for several datasets, particularly datasets based on *organoboron* splitting of the SMC reaction. Nevertheless, for the majority of the datasets, chemical structural information was important in the yield prediction.

From analysis of poorly predicted reaction data points for the two HTE datasets, we revealed possible explanations for the prediction failure by data-driven models. For the BHC reaction datasets, the observed yields of the reactions containing a specific combination of one of three aryl halides (3-chloropyridine, 3-iodopyridine, or 3-bromopyridine) and 4-phenylisoxazole as an additive showed a completely different distribution from the other combinations for the same additive. Because no pairs of aryl halides and additives showed the same specific trend in the training dataset, inferring the structure–reactivity relationship using any data-driven approach based on the structural formula seems to be difficult.

## Data Availability

The code of *MPNN-Transformer*, the BHC reaction datasets, the SMC reaction datasets, the pretrained MPNN, and the results described in this work are available at https://github.com/sa-akinori/Graph_based_transformer_for_yield_prediction_of_HTE.

## Supporting Information Available

The distributions of the observed yields for the *Test1–Test4* datasets of the BHC reaction (**Figure S1**) and *Test1–Test12* datasets of the SMC reaction (**Figure S2**), observed yield versus predicted yield plots for the reactions including benzo[c]isoxazole (**Figure S3**), observed yield versus predicted yield plots for the *Test1–Test12* datasets of the SMC reaction (**Figure S4**), learning curves of the *MPNN-Transformer(3.0m)* models for the SMC reaction datasets (**Figure S5**), comparison of the prediction results of *T5Chem and MPNN-Transformer(3.0m)* for the *Test2* dataset (**Figure S6**), hyper-parameters of the prediction models for comparison (**Table S1**), batch size of the deep learning models for the *Random* datasets (**Table S2**), hyper-parameters of the *MPNN-Transformer* models (**Table S3**), average prediction accuracies (*R*^2^) for the *Random* datasets, *Test1–Test4* datasets, and *sTest1–sTest20* datasets of the BHC reaction (**Tables S4–S6**), prediction accuracies (*R*^2^) for the *sTest1–sTest20* datasets of the BHC reaction (**Table S7**), average prediction accuracies (*R*^2^) for the *Random* datasets of the SMC reaction (**Table S8**), prediction accuracies (*R*^2^) for the *Test1* and *Test3–Test5* datasets of the SMC reaction (**Table S9**), average prediction accuracies (*R*^2^) for the *Test1–Test12* datasets of the SMC reaction (**Table S10**), *p*-values of the Welch’s *t* test of *R*^2^ between *XGBoost* and *MPNN-Transformer(3.0m)* (**Table S11**), average prediction accuracies (*R*^2^) in the additive-out ablation study for the *Test1–Test4* datasets of the BHC reaction (**Tables S12**), prediction accuracies (*R*^2^) in the component-out ablation study for the *Test1–Test6* datasets of the SMC reaction (**Table S13**), average prediction accuracies (*R*^2^) in the component-out ablation study for the *Test1–Test12* datasets of the SMC reaction (**Tables S14**), *p*-values in the Welch’s *t* test of *R*^2^ between *MPNN* and *MPNN(3.0m)* and between *MPNN-Transformer* and *MPNN-Transformer(3.0m)* (**Tables S15** and **S16**). (PDF)
