# Supplementary material for: Chemical Graph-Based Transformer Models for Yield Prediction of High-Throughput Cross-Coupling Reaction Datasets
Source: ACS Omega. 2024 Sep 17;9(39):40907–19. doi: 10.1021/acsomega.4c06113 (PMC11447720; doi:10.1021/acsomega.4c06113)
Supplement: Supplementary file 1 — ao4c06113_si_001.pdf [file ao4c06113_si_001.pdf]

Chemical-Graph-Based Transformer Models for Yield Prediction of High-Throughput Cross-Coupling Reaction Data Sets

Akinori Sato[1], Ryosuke Asahara[2], Tomoyuki Miyao*[1,2]

[1]Data Science Center, Nara Institute of Science and Technology, 8916-5 Takayama-cho, Ikoma, Nara, 630-0192, Japan

[2]Graduate School of Science and Technology, Nara Institute of Science and Technology, 8916-5 Takayama-cho, Ikoma, Nara, 630-0192, Japan

*Corresponding author:

miyao@dsc.naist.jp

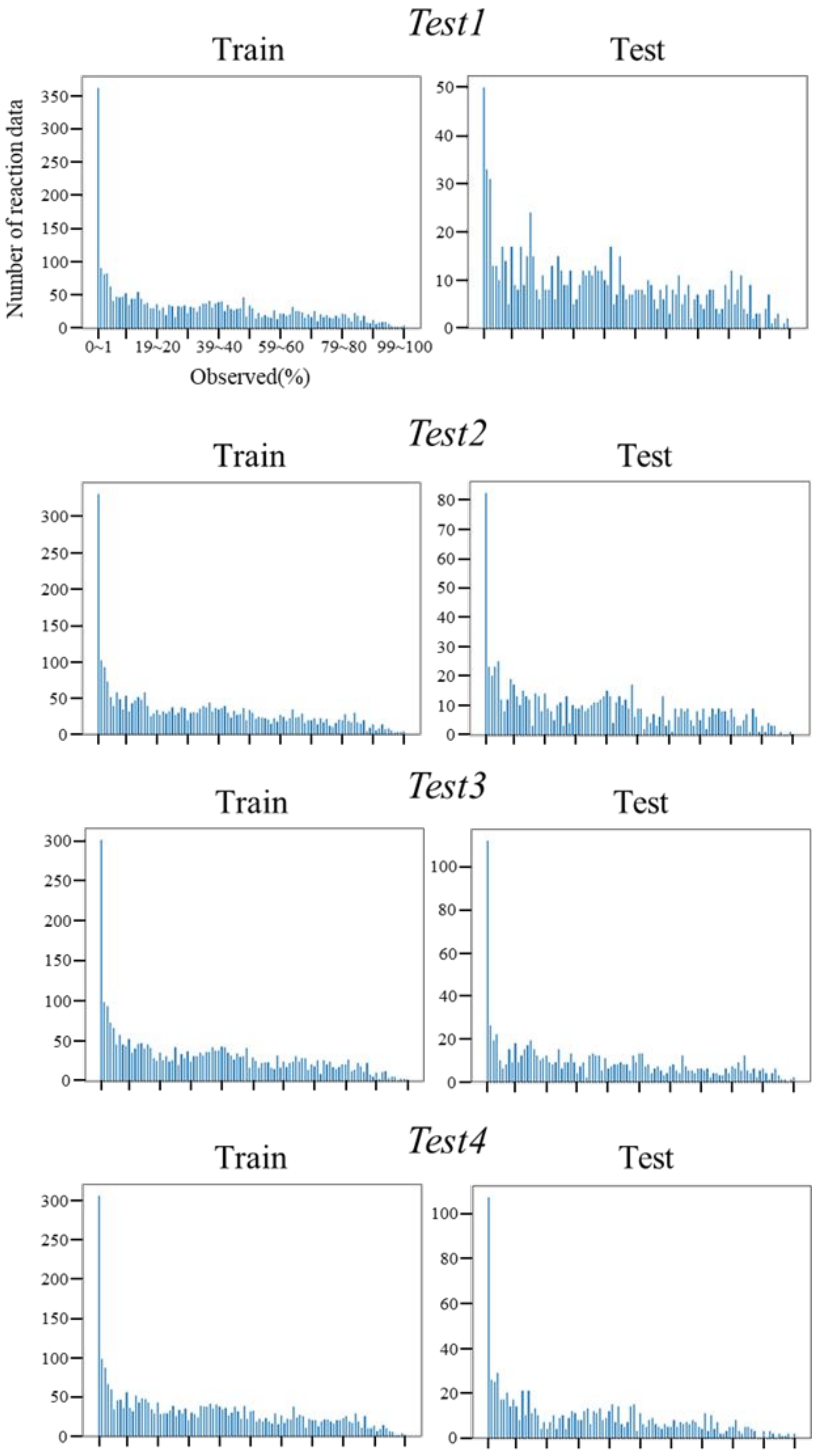

Fig. S1 The distributions of the observed yields for the *Test1-Test4* data sets of the BHC reaction. The horizontal axis is the yield, and the vertical axis is the frequency of reactions. Each bar represents 1% of yield.

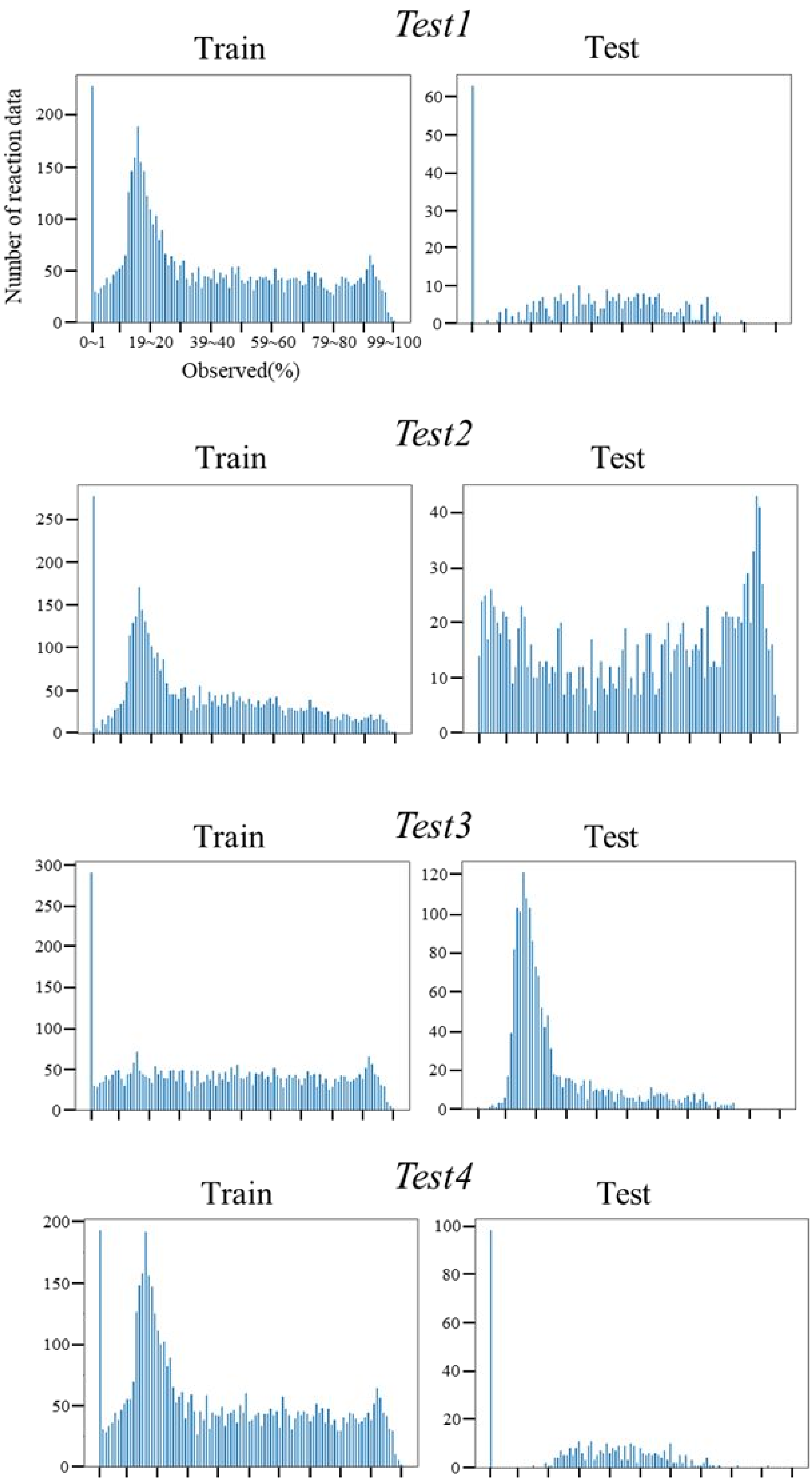
Test1
Train
Test
Number of reaction data
Observed(%)
0~1
19~20
39~40
59~60
79~80
99~100
Test2
Train
Test
Test3
Train
Test
Test4
Train
Test

Test5

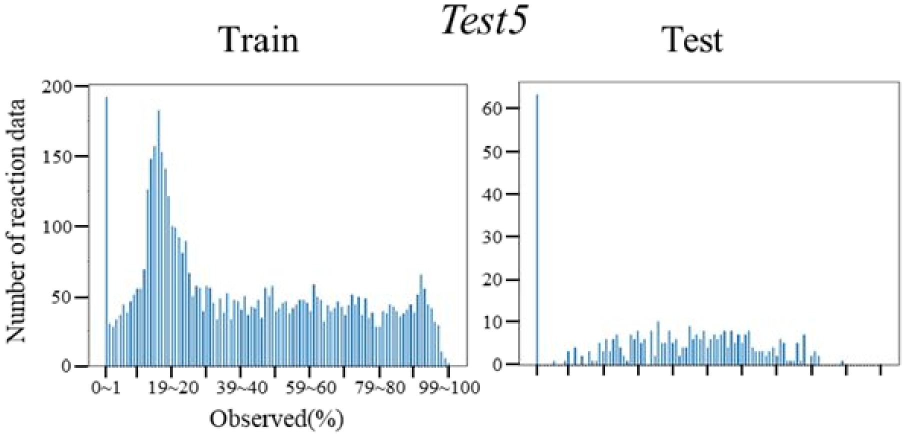

Test6

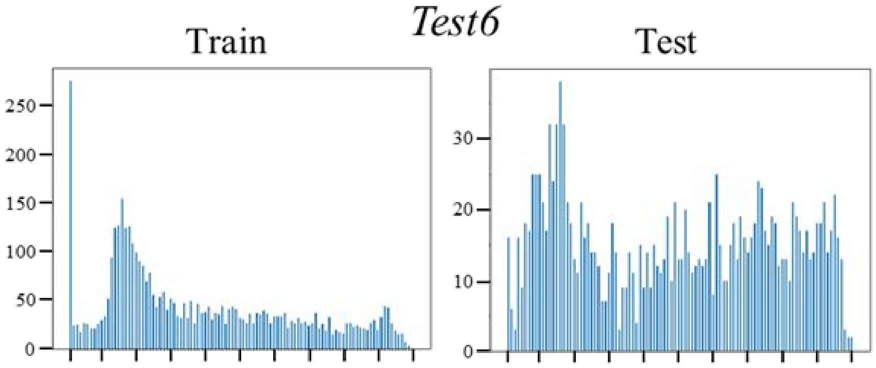

Test7

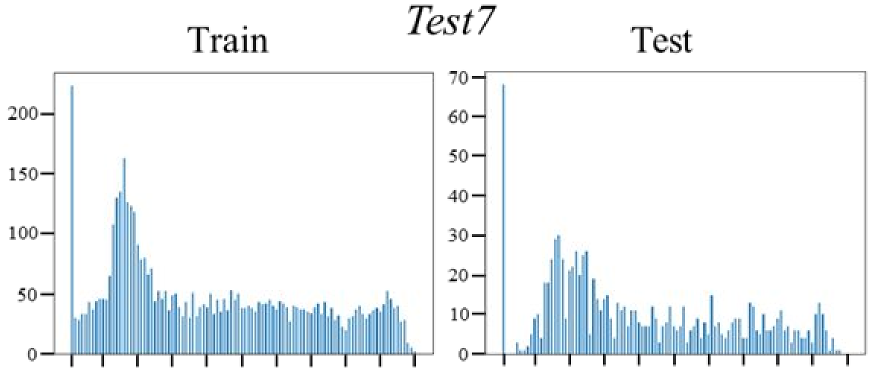

Test8

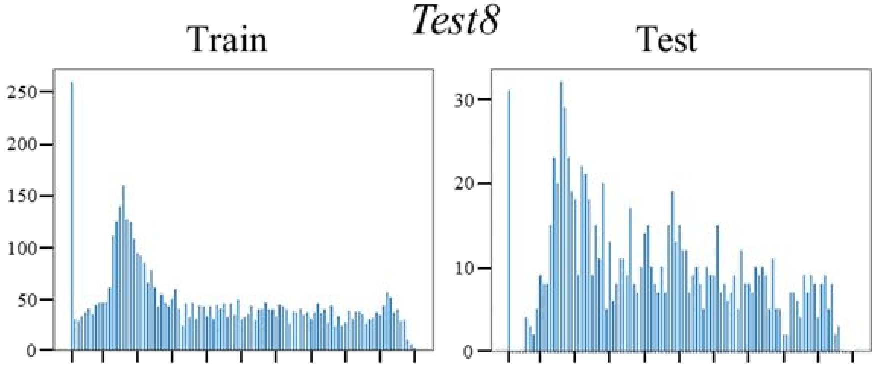

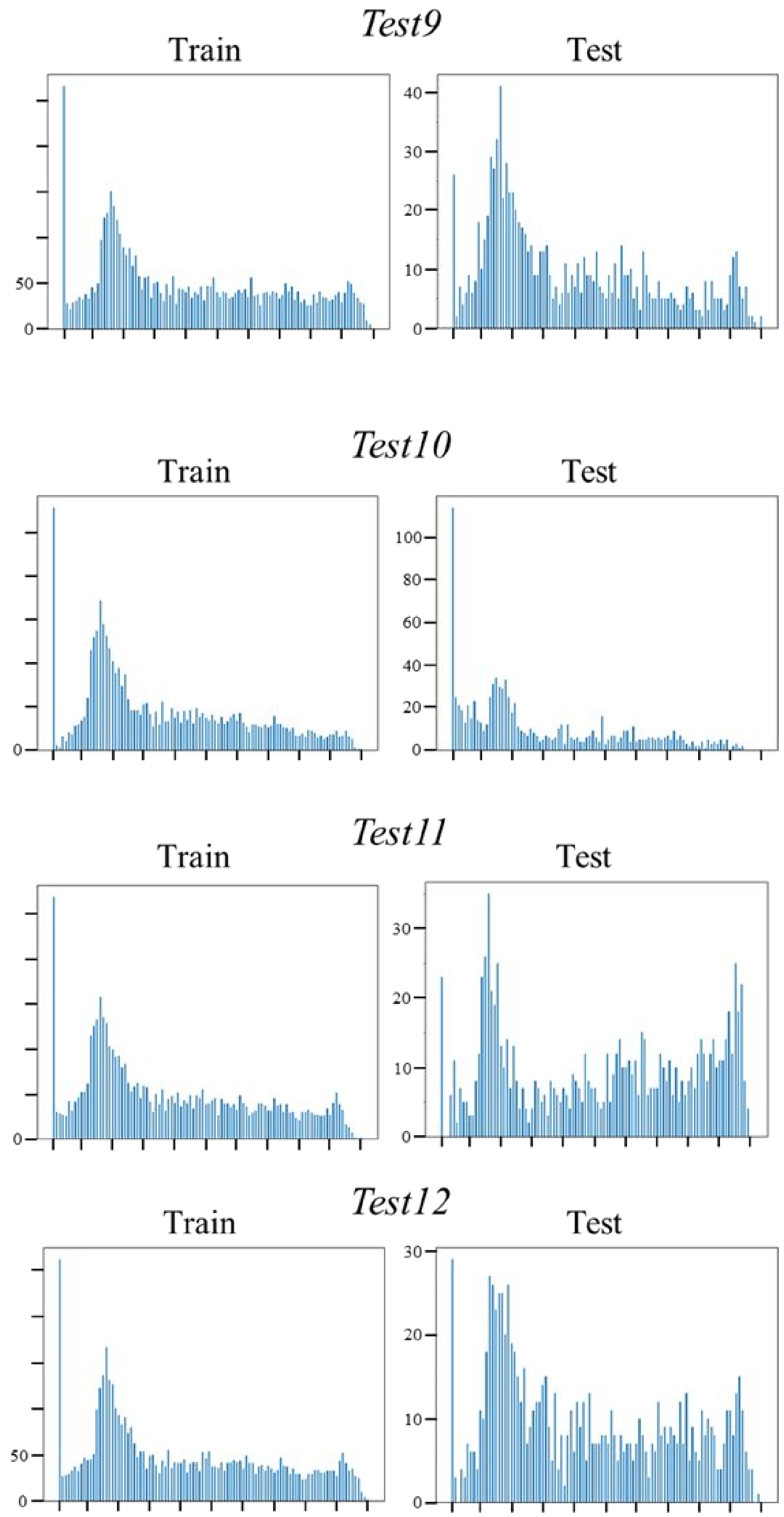

Fig. S2 The distributions of the observed yields for the *Test1-Test12* data sets of the SMC reaction. The horizontal axis is the yield, and the vertical axis is the frequency of reactions. Each bar represents 1% of yield.

Table S1. Hyper-parameters of the prediction models for comparison.

Model	Hyper-parameters	Range (Interval)
Yield-BERT	Pre-trained model	ft, pretrained
	Dropout rate	0.1~0.9 (0.1)
T5Chem	Learning rate	1e-5, 1e-4, 5e-4
	Batch size	16, 32, 64
XGBoost	Max_detph	3~9 (2)
	Min_child_weight	1~5 (2)
	Colsample_bytree	0.6~1.0 (0.1)
	Subsample	0.6~1.0 (0.1)
	Eta	1.0, 0.1, 0.01
	Reg_alpha	1.0, 0.3, 0.1
	Reg_lambda	1.0, 0.3, 0.1
	Learning_rate	0.3, 0.1, 0.01

Multiple values in a cell of the "Range" column were optimized as hyper-parameters. The value in parenthesis represents the interval of the optimized hyperparameter. "Pre-trained model" of *Yield-BERT* in **Table S1** represents two pre-trained models prepared by Schwaller et al.

Table S2. Batch size of the deep learning models for the *Random* data sets.

Dataset	Training : test	Batch size (*Yield-BERT*)	Batch size (*T5Chem*)	Batch size (*MPNN-Transformer*s)
Buchwald-Hartwig	50:50	4, 8, 16	4, 8, 16	8, 16
	30:70	2, 4, 8	4, 8, 16	4, 8
	20:80	2, 4, 8	4, 8, 16	2, 4
	10:90	2, 4, 8	4, 8, 16	1, 2, 4
	5.0:95.0	1, 2, 4	1, 2, 4, 8, 16	1, 2, 4
	2.5:97.5	1, 2, 4	1, 2, 4, 8, 16	1, 2, 4
	1.0:99.0	1, 2, 4	1, 2, 4, 8	1, 2, 4
Suzuki-Miyaura	50:50	4, 8, 16	4, 8, 16	8, 16
	30:70	2, 4, 8	4, 8, 16	4, 8, 16
	20:80	2, 4, 8	4, 8, 16	2, 4, 8
	10:90	2, 4, 8	4, 8, 16	2, 4, 8
	5.0:95.0	1, 2, 4	1, 2, 4, 8	1, 2, 4
	2.5:97.5	1, 2, 4	1, 2, 4, 8	1, 2, 4
	1.0:99.0	1, 2, 4	1, 2, 4, 8	1, 2, 4

Batch size of *Yield-BERT* for the *Random* data set (training : test = 70:30) is the same as the original paper. Batch size of *T5Chem* for the *Random* data set is optimized in **Table S1**. Batch size of *MPNN-Transformer*s for the *Random* data set (training : test = 70:30) is the same in **Table S3**. The other hyper-parameters except for batch size of these models are the same for all data sets.

Table S3. Hyper-parameters of the *MPNN-Transformer* models.

Component	Hyper-parameter	Value
	Batch size	16
	Epochs	200
	Learning rate[b]	1e-4
	Step size of torch.optim.lr_schedule.StepLR	150
	Gamma of torch.optim.lr_schedule.StepLR	0.1
	Loss function	MSELoss
	Optimization method	Adam
MPNN	Learning rate[b]	1e-4
	Dropout rate	0.0
	Number of layers in MLP_h^L	4
	Iterations of message passing L	2
	Number of nodes in hidden layer	400
Transformer encoder	Learning rate	1e-4
	Number of transformer encoder layer N[a]	3, 6, 9
	Number of neurons in FFN	1024
	Number of heads of self-attention	10
	Dropout rate	0.1
MLP	Learning rate	1e-4
	Number of hidden layers[a]	1, 3
	Dropout rate	0.1

[a]Parameters optimized by grid-search using the *Random* data set (training : test = 70:30), where the training data set was further split into 9:1 training and validation data sets. Other hyper-parameter values were determined based on the previous studies. [b]Learning-rate of the pre-trained MPNN sets 1e-5.

Table S4. Average prediction accuracies (R^2) for the *Random* data sets of the BHC reaction.

Training:test	70:30	50:50	30:70	20:80	10:90	5:90	2.5:97.5	1.0:99.0
One-hot-RF	0.89 (0.01)	0.87 (0.01)	0.84 (0.01)	0.81 (0.01)	0.74 (0.02)	0.64 (0.04)	0.49 (0.09)	0.25 (0.11)
Random-RF	0.92 (0.01)	0.90 (0.01)	0.86 (0.01)	0.83 (0.01)	0.76 (0.02)	0.64 (0.04)	0.53 (0.03)	0.27 (0.10)
Yield-BERT	0.96 (0.01)	0.91 (0.01)	0.86 (0.01)	0.84 (0.01)	0.76 (0.03)	0.64 (0.07)	0.44 (0.10)	0.20 (0.10)
T5Chem	0.97 (0.00)	0.96 (0.01)	0.92 (0.01)	0.88 (0.02)	0.80 (0.02)	0.72 (0.03)	0.59 (0.06)	0.31 (0.13)
XGBoost	0.95 (0.00)	0.94 (0.00)	0.90 (0.01)	0.88 (0.01)	0.82 (0.01)	0.76 (0.02)	0.65 (0.03)	0.44 (0.10)
MPNN-Transformer	0.97 (0.00)	0.95 (0.00)	0.92 (0.01)	0.88 (0.01)	0.80 (0.02)	0.70 (0.02)	0.59 (0.05)	0.38 (0.09)
MPNN-Transformer(3.0m)	0.97 (0.00)	0.96 (0.00)	0.93 (0.01)	0.90 (0.01)	0.82 (0.01)	0.74 (0.02)	0.61 (0.05)	0.15 (0.09)

For each model and data set, the average (standard deviation) of the R^2 value for the *Random* data sets of the BHC reaction using the 50 different models initialized with 50 different random is reported.

Table S5. Average prediction accuracies (R^2) for the *Test1–Test4* data sets of the BHC reaction.

	Test1	*Test2*	*Test3*	*Test4*
One-hot-RF	0.69 (0.00)	0.67 (0.00)	0.50 (0.00)	0.48 (0.00)
Random-RF	0.69 (0.00)	0.82 (0.00)	0.51 (0.00)	0.42 (0.00)
Yield-BERT	0.82 (0.02)	0.81 (0.03)	0.70 (0.04)	0.43 (0.08)
T5Chem	0.80 (0.02)	0.87 (0.00)	0.72 (0.03)	0.49 (0.07)
XGBoost	0.88 (0.00)	0.89 (0.00)	0.60 (0.00)	0.58 (0.00)
MPNN-Transformer	0.86 (0.02)	0.84 (0.05)	0.55 (0.07)	0.59 (0.08)
MPNN-Transformer(3.0m)	0.84 (0.03)	0.88 (0.01)	0.70 (0.04)	0.58 (0.04)

For each model and data set, the average (standard deviation) of the R^2 value for the *Test1–Test4* data sets of the BHC reaction using the 50 different models initialized with 50 different random seeds is reported.

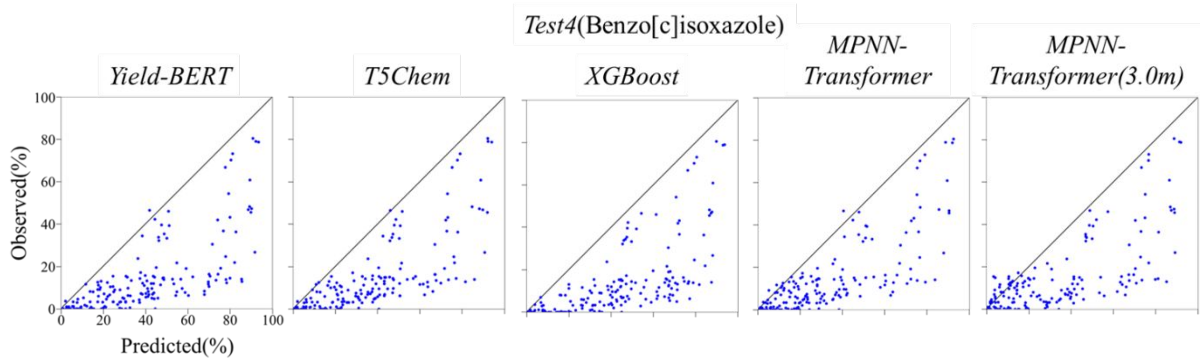

Fig. S3 Observed yield versus predicted yield plots for the reactions including benzo[c]isoxazole.

Table S6. Average prediction accuracies (R^2) for the *sTest1–sTest20* data sets of the BHC reaction.

	sTest1	*sTest2*	*sTest3*	*sTest4*	*sTest5*	*sTest6*	*sTest7*
One-hot-RF	-2.21 (0.09)	-0.13 (0.01)	0.00 (0.01)	-5.01 (0.05)	-8.71 (0.11)	-0.62 (0.04)	-0.26 (0.01)
Random-RF	-7.11 (0.06)	-0.06 (0.02)	-0.14 (0.02)	-0.75 (0.05)	-0.34 (0.09)	-3.95 (0.05)	-0.06 (0.02)
Yield-BERT	-5.94 (0.63)	-0.28 (0.23)	0.38 (0.15)	0.52 (0.10)	-0.89 (0.39)	-0.96 (0.18)	0.71 (0.05)
T5Chem	-3.98 (1.21)	-0.21 (0.33)	0.37 (0.21)	0.61 (0.12)	-1.04 (0.41)	-0.82 (0.27)	0.68 (0.05)
XGBoost	-6.20 (0.08)	0.01 (0.02)	-0.22 (0.04)	0.73 (0.03)	-0.57 (0.04)	-1.43 (0.07)	0.42 (0.01)
MPNN-Transformer	-1.04 (1.33)	0.35 (0.27)	0.04 (0.28)	0.43 (0.32)	-1.21 (0.74)	0.33 (0.48)	0.64 (0.08)
MPNN-Transformer(3.0m)	-1.73 (0.88)	0.42 (0.11)	0.56 (0.12)	0.53 (0.13)	-0.66 (0.23)	0.28 (0.34)	0.69 (0.07)

	sTest8	*sTest9*	*sTest10*	*sTest11*	*sTest12*	*sTest13*	*sTest14*
One-hot-RF	-0.02 (0.01)	-5.10 (0.06)	-8.58 (0.10)	0.03 (0.01)	-0.35 (0.02)	-0.19 (0.02)	-5.59 (0.03)
Random-RF	-0.01 (0.04)	-0.73 (0.07)	-0.94 (0.06)	-1.53 (0.02)	0.02 (0.01)	0.12 (0.02)	-1.03 (0.07)
Yield-BERT	-0.37 (0.18)	-0.58 (0.23)	-1.53 (0.77)	-0.53 (0.07)	0.73 (0.05)	-0.01 (0.15)	-0.24 (0.21)
T5Chem	0.04 (0.28)	-0.43 (0.17)	-1.98 (0.81)	-1.18 (0.35)	-0.08 (0.21)	0.60 (0.09)	0.62 (0.13)
XGBoost	0.51 (0.02)	0.53 (0.02)	-0.42 (0.04)	-0.23 (0.04)	0.57 (0.01)	0.42 (0.02)	0.18 (0.02)
MPNN-Transformer	0.66 (0.15)	-0.32 (0.41)	0.33 (0.20)	-1.56 (0.70)	-0.16 (0.36)	0.17 (0.22)	0.51 (0.23)
MPNN-Transformer(3.0m)	0.54 (0.18)	-0.04 (0.36)	-0.12 (0.49)	-0.21 (0.40)	0.62 (0.19)	0.48 (0.21)	0.08 (0.55)

	sTest15	*sTest16*	*sTest17*	*sTest18*	*sTest19*	*sTest20*
One-hot-RF	-9.27 (0.03)	-0.45 (0.06)	-2.84 (0.08)	-4.72 (0.19)	-42.73 (0.12)	-20.34 (0.11)
Random-RF	-1.66 (0.12)	-0.37 (0.05)	0.11 (0.02)	-1.84 (0.13)	-18.93 (0.47)	-8.78 (0.17)
Yield-BERT	-1.13 (0.47)	-3.70 (0.30)	0.29 (0.14)	-11.95 (0.58)	-20.78 (1.10)	-6.66 (1.33)
T5Chem	0.15 (0.21)	-3.35 (0.38)	0.54 (0.06)	-9.92 (0.98)	-18.77 (1.20)	-7.49 (0.91)
XGBoost	-0.67 (0.04)	0.15 (0.03)	0.57 (0.01)	-0.61 (0.11)	-6.17 (0.13)	-0.95 (0.05)
MPNN-Transformer	-0.43 (0.54)	-5.12 (1.45)	0.21 (0.38)	-4.26 (0.88)	-16.18 (3.04)	-1.25 (1.59)
MPNN-Transformer(3.0m)	0.10 (0.52)	-1.12 (0.90)	0.55 (0.08)	-4.50 (1.12)	-16.44 (2.24)	-1.71 (1.32)

For each model and data set, the average (standard deviation) of the R^2 value for the *sTest1–sTest20* data sets of BHC reaction using the 50 different models initialized with 50 different random seeds is reported.

Table S7. Prediction accuracies (R^2) for the *sTest1–sTest20* data sets of the BHC reaction.

	sTest1	*sTest2*	*sTest3*	*sTest4*	*sTest5*	*sTest6*	*sTest7*
One-hot-RF	-2.21 (0.01)	-0.14 (0.00)	-0.01 (0.00)	-5.02 (0.01)	-8.72 (0.02)	-0.62 (0.01)	-0.26 (0.00)
Random-RF	-7.11 (0.01)	-0.06 (0.01)	-0.14 (0.01)	-0.73 (0.03)	-0.34 (0.01)	-3.94 (0.00)	-0.06 (0.00)
Yield-BERT	-5.84 (0.24)	-0.25 (0.09)	**0.43 (0.04)**	0.61 (0.03)	-0.60 (0.06)	-0.89 (0.08)	**0.73 (0.02)**
T5Chem	-3.61 (0.34)	-0.17 (0.07)	**0.50 (0.09)**	**0.74 (0.03)**	-0.66 (0.09)	-0.71 (0.06)	0.69 (0.01)
XGBoost	-6.20 (0.02)	**0.01 (0.00)**	-0.22 (0.01)	**0.73 (0.01)**	-0.56 (0.02)	-1.43 (0.01)	0.42 (0.00)
MPNN-Transformer	-0.47 (0.30)	**0.42 (0.06)**	0.20 (0.03)	**0.65 (0.05)**	-0.73 (0.33)	**0.65 (0.05)**	**0.72 (0.03)**
MPNN-Transformer(3.0m)	-1.49 (0.37)	**0.44 (0.02)**	**0.64 (0.03)**	0.58 (0.06)	-0.43 (0.06)	**0.42 (0.15)**	**0.72 (0.01)**

	sTest8	*sTest9*	*sTest10*	*sTest11*	*sTest12*	*sTest13*	*sTest14*
One-hot-RF	-0.02 (0.00)	-5.11 (0.03)	-8.54 (0.04)	**0.02 (0.00)**	-0.35 (0.00)	-0.19 (0.01)	-5.61 (0.01)
Random-RF	0.00 (0.01)	-0.75 (0.02)	-0.91 (0.04)	-1.51 (0.01)	0.02 (0.01)	0.12 (0.00)	-1.03 (0.01)
Yield-BERT	-0.33 (0.06)	-0.51 (0.05)	-1.26 (0.15)	-0.51 (0.05)	**0.75 (0.02)**	0.03 (0.09)	-0.19 (0.08)
T5Chem	0.12 (0.12)	-0.37 (0.02)	-1.83 (0.26)	-1.08 (0.04)	-0.06 (0.04)	**0.66 (0.02)**	**0.74 (0.03)**
XGBoost	**0.51 (0.01)**	**0.53 (0.00)**	-0.41 (0.01)	-0.23 (0.01)	**0.57 (0.00)**	**0.42 (0.01)**	**0.18 (0.00)**
MPNN-Transformer	**0.74 (0.03)**	-0.15 (0.12)	**0.53 (0.05)**	-1.43 (0.19)	-0.10 (0.08)	0.21 (0.03)	**0.72 (0.06)**
MPNN-Transformer(3.0m)	**0.61 (0.08)**	**0.06 (0.12)**	**0.09 (0.18)**	0.00 (0.18)	**0.72 (0.08)**	**0.63 (0.04)**	0.16 (0.24)

	sTest15	*sTest16*	*sTest17*	*sTest18*	*sTest19*	*sTest20*
One-hot-RF	-9.27 (0.02)	-0.46 (0.02)	-2.84 (0.03)	-4.67 (0.04)	-42.73 (0.07)	-20.30 (0.05)
Random-RF	-1.69 (0.04)	-0.37 (0.01)	0.11 (0.00)	-1.88 (0.03)	-18.93 (0.14)	-8.83 (0.07)
Yield-BERT	-0.97 (0.14)	-3.55 (0.14)	0.33 (0.04)	-11.85 (0.08)	-20.55 (0.34)	-6.39 (0.28)
T5Chem	**0.32 (0.05)**	-3.13 (0.15)	**0.57 (0.02)**	-9.75 (0.22)	-18.55 (0.37)	-7.30 (0.20)
XGBoost	-0.67 (0.01)	**0.15 (0.01)**	**0.58 (0.00)**	-0.60 (0.04)	-6.17 (0.04)	-0.95 (0.01)
MPNN-Transformer	-0.28 (0.17)	-4.50 (0.60)	0.33 (0.09)	-3.90 (0.21)	-15.49 (0.37)	-0.46 (0.36)
MPNN-Transformer(3.0m)	**0.48 (0.10)**	-0.76 (0.39)	**0.60 (0.01)**	-4.22 (0.44)	-16.19 (1.28)	-1.22 (0.48)

For each model and data set, the average (standard deviation) of the R^2 value for the s*Test1*–*sTest20* data sets of the BHC reaction using the five ensemble models is reported. For each test data set, the three highest R^2 values are highlighted in bold, unless $R^2 < 0$.

Table S8. Average prediction accuracies (R^2) for the *Random* data sets of the SMC reaction.

	70:30	50:50	30:70	20:80	10:90	5.0:95.0	2.5:97.5	1.0:99.0
One-hot-RF	0.84 (0.01)	0.82 (0.00)	0.78 (0.01)	0.75 (0.01)	0.68 (0.01)	0.61 (0.03)	0.50 (0.04)	0.23 (0.08)
Random-RF	0.84 (0.01)	0.82 (0.01)	0.78 (0.00)	0.75 (0.01)	0.68 (0.01)	0.60 (0.01)	0.46 (0.06)	0.27 (0.05)
Yield-BERT	0.77 (0.01)	0.74 (0.01)	0.68 (0.02)	0.62 (0.03)	0.51 (0.04)	0.38 (0.04)	0.21 (0.11)	0.13 (0.13)
T5Chem	0.86 (0.01)	0.84 (0.01)	0.79 (0.01)	0.75 (0.01)	0.63 (0.02)	0.50 (0.03)	0.32 (0.07)	0.08 (0.13)
XGBoost	0.86 (0.00)	0.85 (0.00)	0.81 (0.00)	0.77 (0.01)	0.70 (0.01)	0.62 (0.01)	0.51 (0.04)	0.31 (0.07)
MPNN-Transformer	0.87 (0.01)	0.85 (0.01)	0.79 (0.01)	0.74 (0.01)	0.64 (0.02)	0.52 (0.02)	0.33 (0.04)	0.03 (0.15)
MPNN-Transformer(3.0m)	0.87 (0.01)	0.83 (0.01)	0.77 (0.01)	0.74 (0.01)	0.67 (0.02)	0.56 (0.01)	0.38 (0.04)	0.05 (0.14)

For each model and data set, the average (standard deviation) of the R^2 value for the *Random* data sets of the SMC reaction using the 50 different models initialized with 50 different random seeds is reported.

Table S9. Prediction accuracies (R^2) for the *Test1 and Test3-Test5* data sets of the SMC reaction.

	Test1	*Test3*	*Test4*	*Test5*
One-hot-RF	0.06 (0.00)	-4.21 (0.00)	0.27 (0.00)	-0.26 (0.00)
Random-RF	0.38 (0.00)	-3.99 (0.00)	0.32 (0.00)	-0.17 (0.01)
Yield-BERT	0.21 (0.05)	-3.46 (0.17)	0.00 (0.01)	-0.86 (0.06)
T5Chem	0.17 (0.04)	-3.34 (0.16)	0.07 (0.08)	-0.72 (0.16)
XGBoost	0.24 (0.01)	-0.15 (0.00)	0.21 (0.00)	0.10 (0.00)
MPNN-Transformer	0.28 (0.06)	-4.25 (0.10)	-0.15 (0.01)	-0.81 (0.12)
MPNN-Transformer(3.0m)	0.17 (0.06)	-4.85 (0.02)	-0.09 (0.02)	-0.57 (0.02)

For each model and data set, the average (standard deviation) of the R^2 value for the *Test1*, *Test3–Test5* data sets of the SMC reaction using the five ensemble models is reported.

Table S10. Average prediction accuracies (R^2) for the *Test1-Test12* data sets of the SMC reaction.

	Test1	*Test2*	*Test3*	*Test4*	*Test5*	*Test6*
One-hot-RF	0.06 (0.01)	-0.43 (0.01)	-4.21 (0.01)	0.27 (0.00)	-0.26 (0.01)	-0.37 (0.01)
Random-RF	0.38 (0.01)	-0.01 (0.03)	-4.00 (0.01)	0.32 (0.00)	-0.17 (0.03)	0.27 (0.03)
Yield-BERT	0.06 (0.15)	0.43 (0.09)	-3.65 (0.57)	-0.06 (0.08)	-1.01 (0.26)	0.57 (0.04)
T5Chem	-0.20 (0.23)	0.59 (0.08)	-3.46 (0.58)	-0.02 (0.13)	-0.97 (0.22)	0.67 (0.02)
XGBoost	0.24 (0.02)	-0.53 (0.01)	-0.15 (0.01)	0.21 (0.01)	0.10 (0.01)	0.08 (0.01)
MPNN-Transformer	0.17 (0.15)	-0.55 (0.06)	-4.33 (0.30)	-0.19 (0.06)	-0.95 (0.23)	0.65 (0.05)
MPNN-Transformer (3.0m)	0.09 (0.14)	-0.09 (0.23)	-4.88 (0.17)	-0.17 (0.08)	-0.63 (0.08)	0.66 (0.09)
	Test7	*Test8*	*Test9*	*Test10*	*Test11*	*Test12*
One-hot-RF	0.00 (0.00)	0.37 (0.00)	0.51 (0.00)	-0.21 (0.00)	0.57 (0.00)	0.64 (0.00)
Random-RF	0.22 (0.01)	0.14 (0.01)	0.40 (0.01)	0.04 (0.02)	0.28 (0.01)	0.43 (0.01)
Yield-BERT	-0.55 (0.10)	0.25 (0.09)	0.00 (0.11)	-0.51 (0.23)	0.19 (0.09)	0.55 (0.04)
T5Chem	-0.10 (0.17)	0.22 (0.06)	0.22 (0.10)	-0.70 (0.17)	0.21 (0.13)	0.57 (0.07)
XGBoost	0.39 (0.01)	0.59 (0.01)	0.65 (0.01)	0.30 (0.01)	0.32 (0.02)	0.66 (0.00)
MPNN-Transformer	0.42 (0.15)	0.51 (0.08)	0.34 (0.07)	-0.61 (0.22)	0.48 (0.08)	0.64 (0.03)
MPNN-Transformer(3.0m)	0.58 (0.03)	0.65 (0.03)	0.34 (0.07)	-0.07 (0.09)	0.57 (0.03)	0.65 (0.04)

For each model and data set, the average (standard deviation) of the R^2 value for the *Test1–Test12* data sets of the SMC reaction using 50 different models initialized with 50 different

seeds.

Table S11. ***p*****-values of the Welch's T-test of** R^2 **between** ***XGBoost*** **and** ***MPNN-Transformer(3.0m)*****.**

Test7	*Test8*	*Test9*	*Test10*	*Test11*	*Test12*
7.04e-8	2.57e-6	4.87e-6	8.33e-7	6.91e-12	2.45e-3

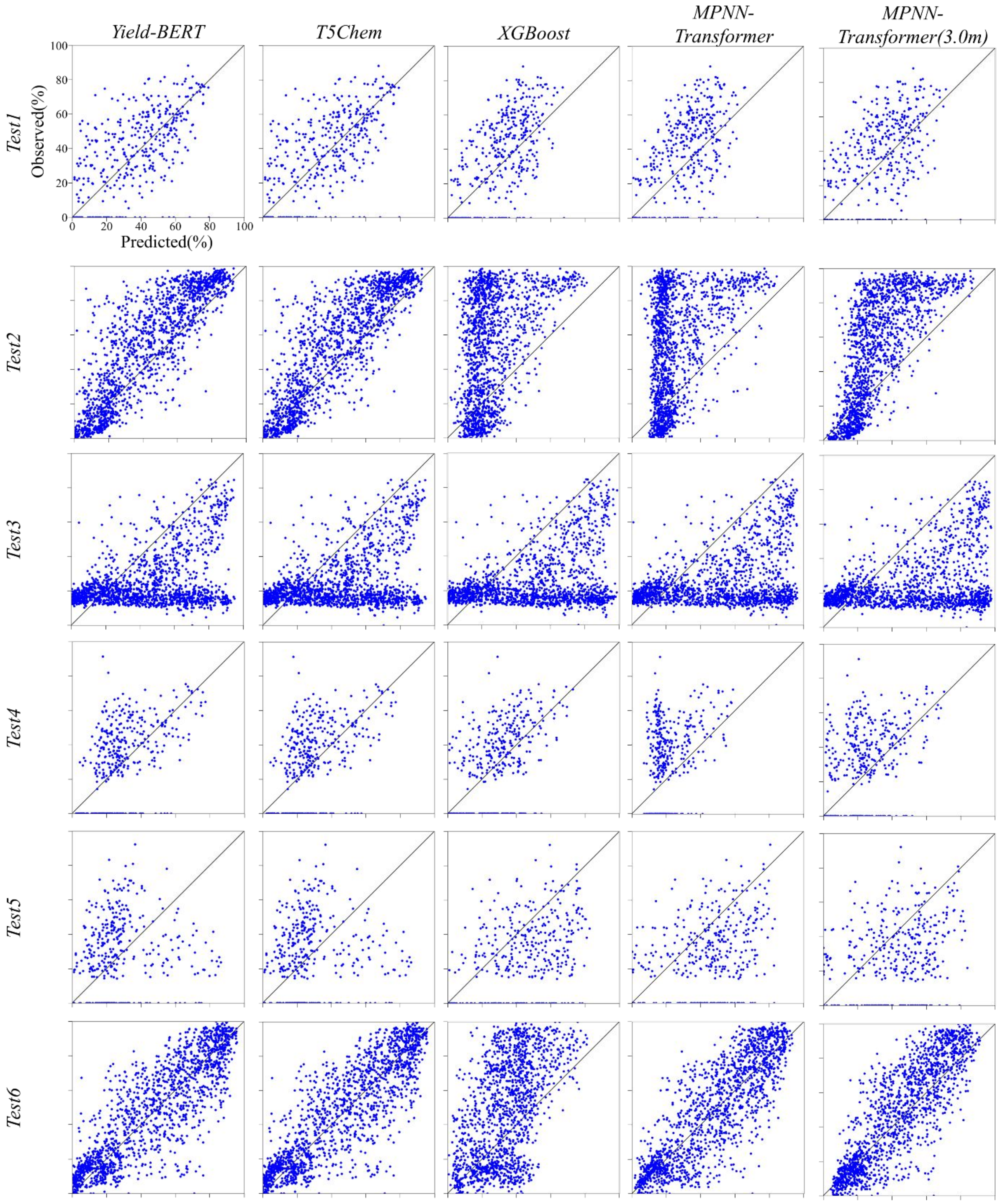

Yield-BERT
T5Chem
XGBoost
MPNN-Transformer
MPNN-Transformer(3.0m)
Test1
Test2
Test3
Test4
Test5
Test6
Observed(%)
Predicted(%)
0
20
40
60
80
100

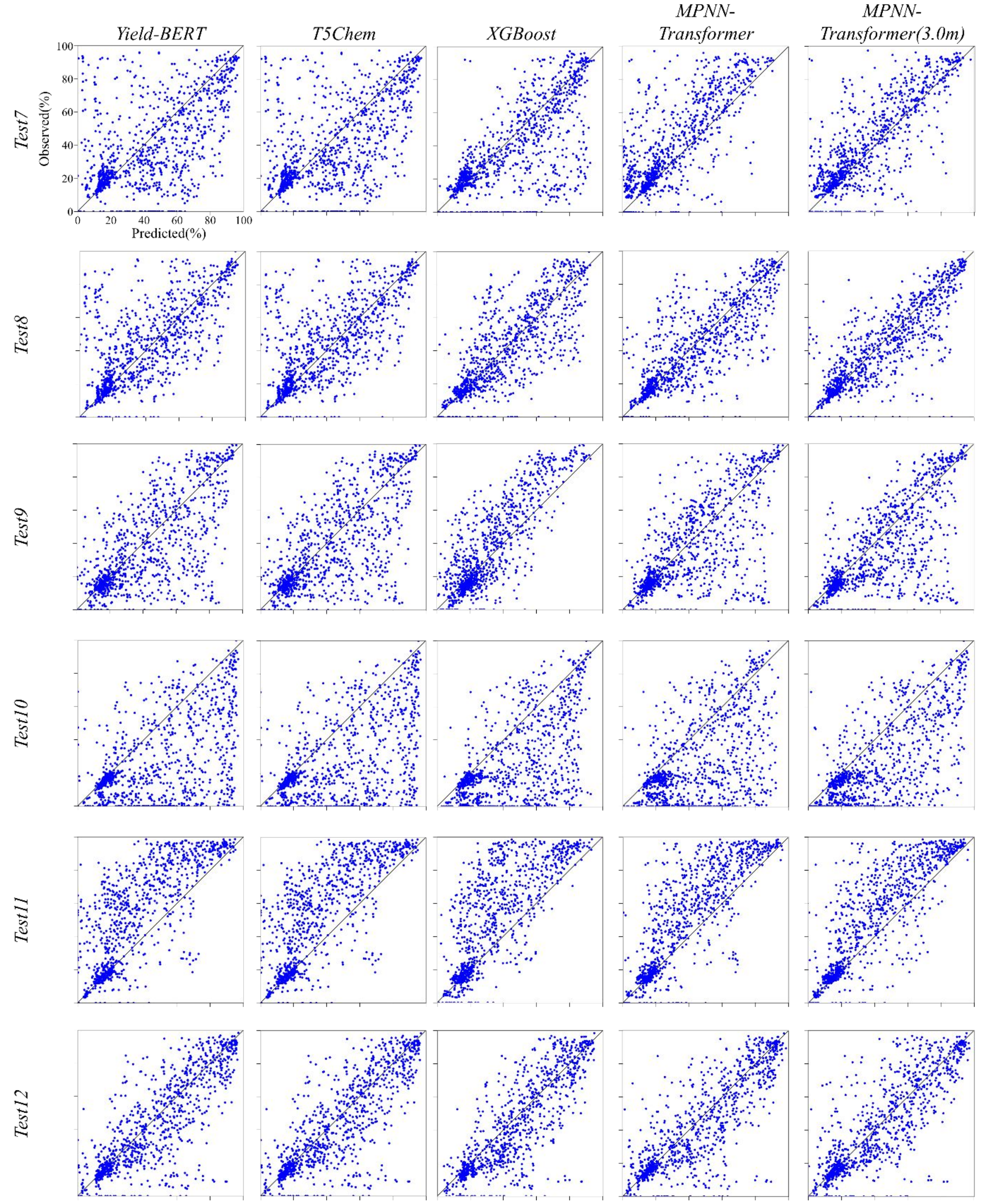

Fig. S4 Observed yield versus predicted yield plots for the *Test1-Test12* data sets of the SMC reaction.

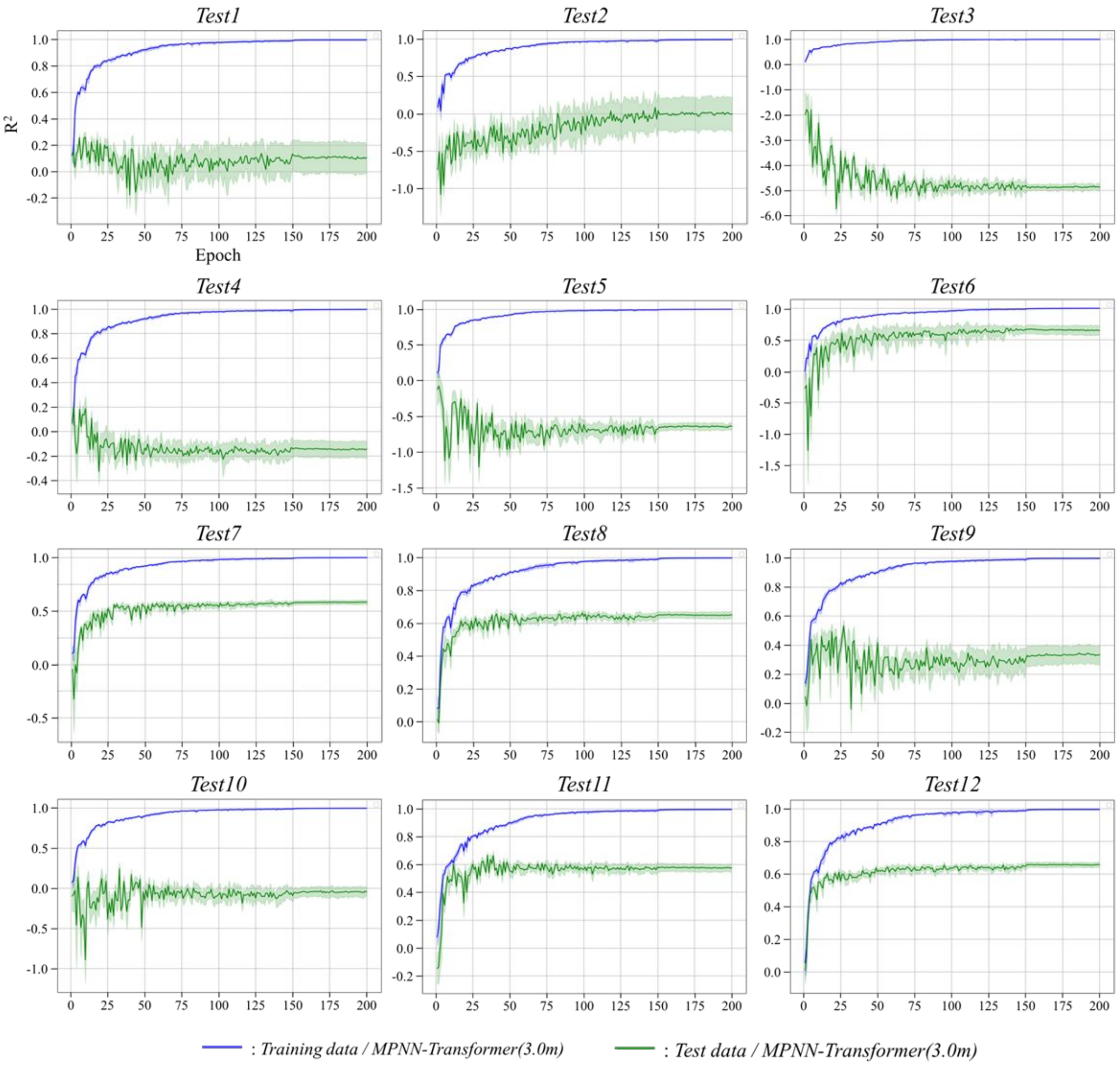

Fig. S5 Learning curves of the *MPNN-Transformer(3.0m)* models for the SMC reaction data sets. The vertical axis represents prediction accuracy (R^2), and the horizontal axis represents the number of epochs. Blue and green lines represent the R^2 of *MPNN-Transformer(3.0m)* for the training and test data, respectively.

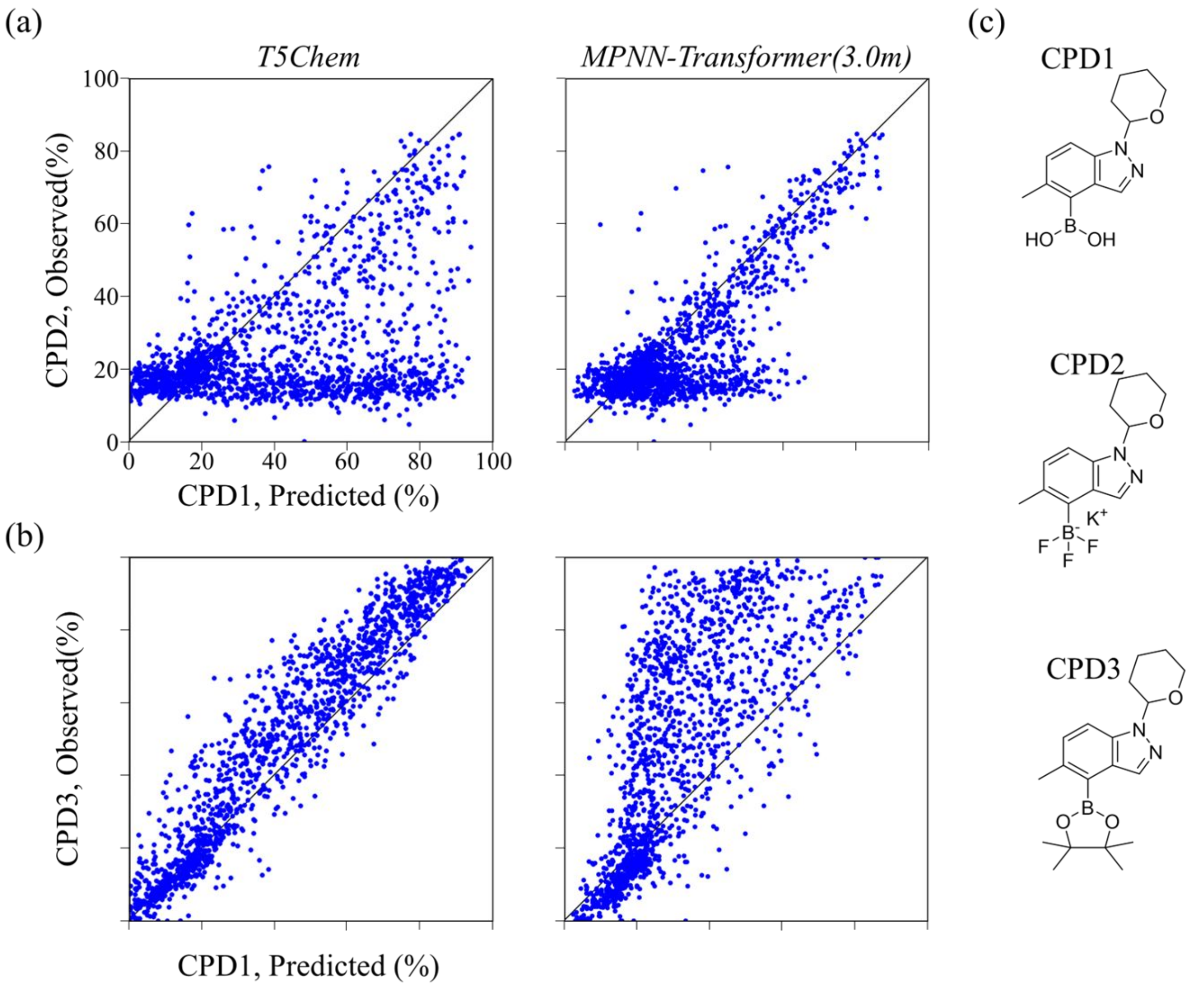

Fig. S6 Comparison of the prediction results of *T5Chem and MPNN-Transformer(3.0m)* for the *Test2* data set. (**a**) Observed yield versus predicted yield plots for the same reaction components except that the two *organoboron* components are shown as scatterplots. The *y* axis of the two plots is the observed yield for the reaction containing CPD2, while the *x* axis is the predicted yield for the reaction containing CPD1. (**b**) Observed yield versus predicted yield plots for the same reaction components except that the two *organoboron* components are shown as scatterplots. The *y* axis of the two plots is the observed yield for the reaction containing CPD3, while the *x* axis is the predicted yield for the reaction containing CPD1. (**c**) Structural formulas of CPD1–CPD3.

Table S12. Average prediction accuracies (R^2) in the additive-out ablation study for the *Test1–Test4* data sets of the BHC reaction.

	Test1	*Test2*	*Test3*	*Test4*
MPNN	0.93<br>(0.01)	0.89<br>(0.01)	0.69<br>(0.03)	0.51<br>(0.03)
MPNN(3.0m)	0.86<br>(0.01)	0.88<br>(0.01)	0.73<br>(0.01)	0.62<br>(0.01)
Transformer	0.90<br>(0.02)	0.90<br>(0.01)	0.59<br>(0.05)	0.39<br>(0.07)
Transformer(random)	0.55<br>(0.01)	0.60<br>(0.01)	0.21<br>(0.01)	0.28<br>(0.02)
MPNN-Transformer	0.86<br>(0.02)	0.84<br>(0.05)	0.55<br>(0.07)	0.59<br>(0.08)
MPNN-Transformer(3.0m)	0.84<br>(0.03)	0.88<br>(0.01)	0.70<br>(0.04)	0.58<br>(0.04)

For each model and data set, the average (standard deviation) of the R^2 value for the *Test1–Test4* data sets of the BHC reaction using 50 different models initialized with 50 different seeds.

Table S13. Prediction accuracies (R^2) in the component-out ablation study for the *Test1*–*Test6* data sets of the SMC reaction.

	Test1	*Test2*	*Test3*	*Test4*	*Test5*	*Test6*
MPNN	0.23 (0.01)	-0.50 (0.00)	-4.70 (0.00)	-0.06 (0.00)	-1.35 (0.01)	0.58 (0.05)
MPNN(3.0m)	0.43 (0.00)	0.33 (0.00)	-3.86 (0.00)	0.26 (0.00)	-1.29 (0.01)	0.74 (0.00)
Transformer	0.14 (0.02)	-0.49 (0.01)	-4.39 (0.09)	-0.17 (0.01)	-0.54 (0.01)	0.63 (0.02)
Transformer(random)	-0.40 (0.02)	0.62 (0.01)	-3.82 (0.06)	-0.08 (0.00)	-0.57 (0.02)	0.69 (0.00)
MPNN-Transformer	0.28 (0.06)	-0.54 (0.01)	-4.25 (0.10)	-0.15 (0.01	-0.81 (0.12)	0.72 (0.01)
MPNN-Transformer(3.0m)	0.17 (0.06)	-0.04 (0.06)	-4.85 (0.02)	-0.09 (0.02)	-0.57 (0.02)	0.73 (0.02)

For each model and data set, the average (standard deviation) of the R^2 value for the *Test1*–*Test6* data sets of the SMC reaction using the five ensemble models is reported.

Table S14. Average prediction accuracies (R^2) in the component-out ablation study for the *Test1–Test12* data sets of the SMC reaction.

	Test1	*Test2*	*Test3*	*Test4*	*Test5*	*Test6*
MPNN	0.17 (0.09)	-0.51 (0.04)	-4.72 (0.14)	-0.08 (0.03)	-1.39 (0.21)	0.56 (0.05)
MPNN(3.0m)	0.41 (0.02)	0.32 (0.05)	-3.89 (0.12)	0.23 (0.03)	-1.31 (0.11)	0.73 (0.01)
Transformer	0.09 (0.11)	-0.50 (0.12)	-4.51 (0.36)	-0.22 (0.07)	-0.63 (0.11)	0.55 (0.16)
Transformer (random)	-0.47 (0.13)	0.59 (0.04)	-4.03 (0.47)	-0.13 (0.06)	-0.65 (0.16)	0.66 (0.02)
MPNN-Transformer	0.17 (0.15)	-0.55 (0.06)	-4.33 (0.30)	-0.19 (0.06)	-0.95 (0.23)	0.65 (0.05)
MPNN-Transformer(3.0m)	0.09 (0.14)	-0.09 (0.23)	-4.88 (0.17)	-0.17 (0.08)	-0.63 (0.08)	0.66 (0.09)
	Test7	*Test8*	*Test9*	*Test10*	*Test11*	*Test12*
MPNN	0.27 (0.09)	0.44 (0.02)	0.33 (0.03)	-0.92 (0.07)	0.11 (0.07)	0.54 (0.03)
MPNN(3.0m)	0.48 (0.04)	0.57 (0.03)	0.34 (0.02)	-0.60 (0.12)	0.18 (0.04)	0.45 (0.03)
Transformer	0.47 (0.05)	0.57 (0.04)	0.37 (0.06)	0.07 (0.03)	0.53 (0.06)	0.60 (0.05)
Transformer (random)	-0.05 (0.12)	-0.02 (0.10)	0.54 (0.06)	-0.48 (0.18)	0.54 (0.01)	0.35 (0.11)
MPNN-Transformer	0.42 (0.15)	0.51 (0.08)	0.34 (0.07)	-0.61 (0.22)	0.48 (0.08)	0.64 (0.03)
MPNN-Transformer(3.0m)	0.58 (0.03)	0.65 (0.03)	0.34 (0.07)	-0.07 (0.09)	0.57 (0.03)	0.65 (0.04)

For each model and data set, the average (standard deviation) of the R^2 value for the *Test1–Test12* data sets of the SMC reaction using 50 different models initialized with 50 different seeds.

Table S15. ***p*-values in the Welch's T-test of R^2 between *MPNN* and *MPNN(3.0m)***

Test7	*Test8*	*Test9*	*Test11*	*Test12*
3.08e-8	6.05e-10	0.27	1.74e-5	2.52e-11

Table S16. ***p*-values in the Welch's T-test of R^2 between *MPNN-Transformer* and *MPNN-Transformer(3.0m)***

Test7	*Test8*	*Test9*	*Test11*	*Test12*
1.15e-4	1.65e-5	0.50	1.29e-3	1.46e-4
